# Characterizing and quantifying low-value diagnostic imaging internationally: a scoping review

**DOI:** 10.1186/s12880-022-00798-2

**Published:** 2022-04-21

**Authors:** Elin Kjelle, Eivind Richter Andersen, Arne Magnus Krokeide, Lesley J. J. Soril, Leti van Bodegom-Vos, Fiona M. Clement, Bjørn Morten Hofmann

**Affiliations:** 1 Institute for the Health Sciences, The Norwegian University of Science and Technology (NTNU) at Gjøvik, NTNU Gjøvik, Postbox 191, 2802 Gjøvik, Norway; 2 Department of Community Health Sciences and The Health Technology Assessment Unit, O’Brien Institute for Public Health, University of Calgary, 3280 Hospital Dr NW, Calgary, AB T2N 4Z6 Canada; 3 Medical Decision Making, Department of Biomedical Data Sciences, Leiden University Medical Center, P.O. Box 9600, 2300 RC Leiden, The Netherlands; 4 Centre of Medical Ethics, The University of Oslo, Blindern, Postbox 1130, 0318 Oslo, Norway

## Abstract

**Background:**

Inappropriate and wasteful use of health care resources is a common problem, constituting 10–34% of health services spending in the western world. Even though diagnostic imaging is vital for identifying correct diagnoses and administrating the right treatment, low-value imaging—in which the diagnostic test confers little to no clinical benefit—is common and contributes to inappropriate and wasteful use of health care resources. There is a lack of knowledge on the types and extent of low-value imaging. Accordingly, the objective of this study was to identify, characterize, and quantify the extent of low-value diagnostic imaging examinations for adults and children.

**Methods:**

A scoping review of the published literature was performed. Medline-Ovid, Embase-Ovid, Scopus, and Cochrane Library were searched for studies published from 2010 to September 2020. The search strategy was built from medical subject headings (Mesh) for Diagnostic imaging/Radiology OR Health service misuse/Medical overuse OR Procedures and Techniques Utilization/Facilities and Services Utilization. Articles in English, German, Dutch, Swedish, Danish, or Norwegian were included.

**Results:**

A total of 39,986 records were identified and, of these, 370 studies were included in the final synthesis. Eighty-four low-value imaging examinations were identified. Imaging of atraumatic pain, routine imaging in minor head injury, trauma, thrombosis, urolithiasis, after thoracic interventions, fracture follow-up and cancer staging/follow-up were the most frequently identified low-value imaging examinations. The proportion of low-value imaging varied between 2 and 100% inappropriate or unnecessary examinations.

**Conclusions:**

A comprehensive list of identified low-value radiological examinations for both adults and children are presented. Future research should focus on reasons for low-value imaging utilization and interventions to reduce the use of low-value imaging internationally.

*Systematic review registration*: PROSPERO: CRD42020208072.

**Supplementary Information:**

The online version contains supplementary material available at 10.1186/s12880-022-00798-2.

## Background

The use of health care and health care expenditures are increasing in most countries [1]. According to the Organization for Economic Co-operation and Development (OECD) 10–34% of health service spending is inappropriate and wasteful use of health care resources [2]. Diagnostic imaging is a health care resource aiding the physician in identifying correct diagnoses and administering the right treatment for the right patient at the right time [3]. However, imaging services can also be inappropriately used or be of low clinical value. While inappropriate imaging is characterized by not being in accordance with professional norms and guidelines, low-value care is defined as services that provide little or no benefit to patients, have potential to cause harm, incur unnecessary cost to patients, or waste limited healthcare resources. Diagnostic imaging would be of low-value when the examination has little or no impact on the management of the individual patient, thus in a societal perspective increasing costs and constituting an unnecessary risk to patients due to exposure to ionizing radiation [4] and/or contrast media [5]. Earlier research found that 20–50% of radiological examinations are overused, however, this rate varies between and within countries [2, 6–8]. Recommendations and guidelines such as the National Institute for Health and Care Excellence’s (NICE’s) “Do-not-do list,” iRefer, iGuide and the international Choosing Wisely campaign have been introduced to reduce overutilization in health care and reduce low-value care, including diagnostic imaging [9–11]. So far, the impact of such efforts is reportedly low, as patient expectations of advanced diagnostic tests, lack of knowledge among health care professionals on the right use of imaging, established clinical practice, fear of malpractice, and fee-for-service reimbursement systems continue to drive the use of low-value care [6, 12–16]. Knowledge about low-value imaging in terms of characteristics, quantities and contexts is warranted to enable adequate prioritizing of resource utilization and designing de-implementation initiatives. A recent systematic review previously estimated the prevalence of low-value diagnostic testing, which included some radiological services, but did not provide a complete overview of which diagnostic imaging examinations that may be regarded as low-value [17]. Therefore, the objective of this scoping review was to identify, characterize, and quantify the extent of low-value diagnostic imaging examinations.

## Methods

A scoping review was completed in accordance with the Preferred Reporting Items for Systematic Reviews and Meta-analyses (PRISMA) extension for scoping reviews [18]. The protocol for this scoping review is registered on the PROSPERO website (CRD42020208072). Medline-Ovid, Embase-Ovid, Scopus, and Cochrane Library were searched for studies published from January 2010 to September 9, 2020. The search strategy was developed in Medline-Ovid (Table 1) and adapted for the other databases with assistance/support from librarians. Terms were built from medical subject headings (Mesh) for Diagnostic imaging/Radiology OR Health service misuse/Medical overuse OR Procedures and Techniques Utilization/Facilities and Services Utilization with text word synonyms of these terms, and more specific terms not having a Mesh term. Language filters were used to include articles written in English, German, Dutch, Danish, Norwegian, and Swedish. Animal studies were excluded. The complete search strategy is available in Additional file 1.

**Table 1:** Search strategy in Medline (Ovid)

#	Medline (Ovid)
1	Diagnostic imaging/or cardiac imaging techniques/or imaging, three-dimensional/or neuroimaging/or radiography/or radionuclide imaging/or respiratory-gated imaging techniques/or tomography/or ultrasonography/or whole body imaging/
2	exp Radiology/
3	(MRI or x-ray* or xray* or ultrasound* or mammography or ultrasonography or DEXA or DXA or CT or radiograph* or radiolog* or tomography or imaging).tw
4	(CAT adj scan).tw
5	(bone adj scan).tw
6	(Magnetic adj resonance adj imaging).tw
7	1 or 2 or 3 or 4 or 5 or 6
8	exp Health Services Misuse/ or exp Medical Overuse/
9	(Unnecessar* or overuse* or Inappropriate* or waste or wasted or low-value or overdiagn* or overutili* or misuse* or (Low adj value) or unwarrent or redundant).tw
10	(Choosing adj wisely).tw
11	8 or 9 or 10
12	7 and 11
13	Animal/ not (animal/ and human/)
14	12 not 13
15	limit 14 to ((danish or Dutch or English or German or Norwegian or Swedish) and last 10 years)

The search was expanded through a snowballing technique of hand-searching the reference lists of articles included following full-text screening.

### Selection of records

The records were archived using Thomson Reuters EndNote X9.3.3 library and duplicates were removed. All remaining records were transferred to Rayyan QCRI [19] where titles and abstracts were screened by EK, ERA, LvB-V, FC, and BMH for eligibility; 10% of citations were screened by two of the authors as quality assurance. Full-text screening was completed by EK, ERA, AMK LvB-V, LJJS and BMH after a calibration meeting for quality assurance. Disagreements with regards to inclusion or exclusion were resolved through discussion and consensus among the authors.

### Eligibility criteria

The inclusion and exclusion criteria are presented in Table 2. In brief, empirical studies, including randomized controlled trials (RCTs), non-randomized controlled trials, cohort studies, descriptive qualitative studies, case studies, mixed-methods studies, and multi-methods studies assessing the value of radiological examinations for all patient groups were included.

**Table 2:** Inclusion and exclusion criteria

Inclusion criteria	Exclusion criteria
Empirical study	Published before 2010
Value of radiological examination	Patient case report, letter, comment, editorial, guidelines
Identifying low-value/inappropriate diagnostic imaging (radiology)	Mass-screening related studies
Extent/use of low-value diagnostic imaging (radiology)	Dental imaging, optical imaging, thermal imaging, microscopic imaging
RCT, non-randomized controlled trial, cohort study, descriptive study, case studies, mixed-methods, multi-methods	Animal studies, studies on cells/tissue
Studies comparing two or more imaging procedures	Studies where imaging is shown to avoid other inappropriate medical procedures/treatments
English, German, Dutch, Danish, Swedish, or Norwegian language	Image quality evaluation/improvement projects
	Interventions to reduce low-value imaging

### Data extraction and synthesis

Data of the included studies were extracted using a summary table consisting of the following variables: author and year, country, design/methods, population, clinical setting, medical condition, low-value practice, reason for being low-value, alternative to low-value practice, and extent of use (when applicable). EK, ERA, AMK, and BMH extracted data after a calibration meeting where 10 publications were discussed for quality assurance. Narrative synthesis of included articles was completed. Articles were first categorized by adult or pediatric, the imaging modality, type of radiological examination evaluated, and the anatomical area imaged.

## Results

**Fig. 1:**
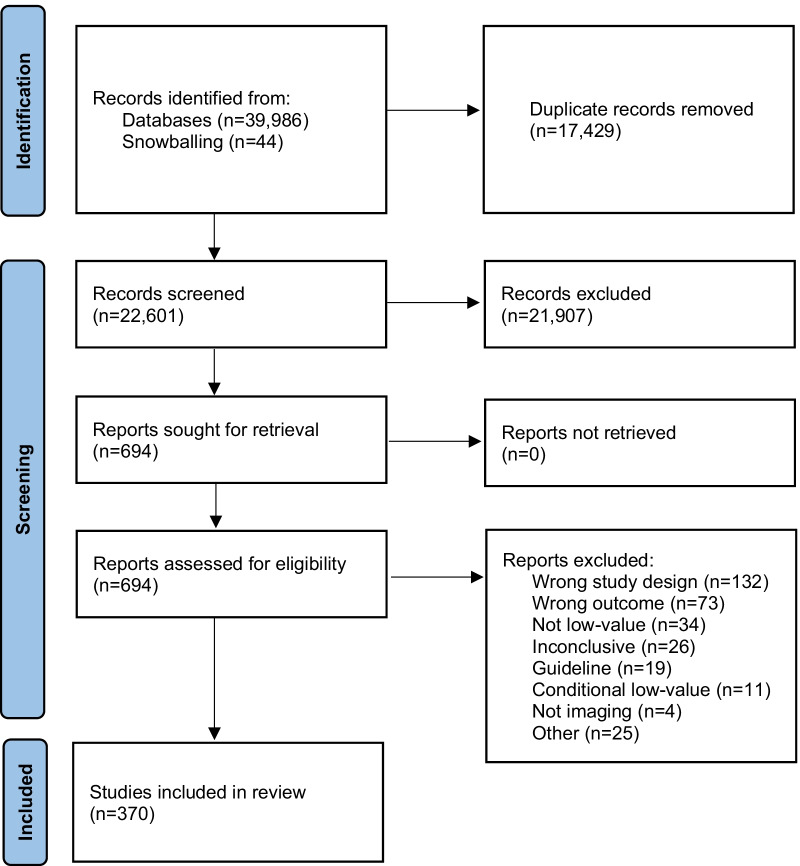
PRISMA flow diagram of the selection process of articles

The electronic database search identified 39,986 records (findings are documented in Additional file 1) and 17,429 duplicates were removed. A total of 22,557 records were screened for titles (and abstracts) in Rayyan QCRI [19] excluding 21,907 records. Through additional searches and snowballing, 44 additional records were found, resulting in 694 articles for full-text assessment. Following full-text screening 324 articles were excluded; an overview of the excluded articles and the reason for exclusion is presented in Additional file 2. Ultimately, 370 studies were included in the final synthesis. A PRISMA flow diagram of the screening and selection process is presented in Fig. 1.

Among the 370 included studies, 84 low-value imaging examinations were identified. Studies were conducted in 35 different countries, with most from the United States (n = 215) and Europe (n = 78). In-hospital imaging was the most common clinical setting (> 65%). Fourteen different study designs were employed among the included studies; most studies were designed as retrospective chart reviews (n = 262), cohort studies (n = 39), and cross-sectional studies (n = 19). Three hundred and eight studies included adult patients, 60 studied pediatric populations, and 2 studies included both adults and children. The characteristics of the included articles are provided in Additional file 3.

### Identified low-value imaging examinations

Low-value diagnostic imaging in adults was evaluated in 264 studies. Across all imaging modalities, low-value use of computed tomography (CT), magnetic resonance imaging (MRI), and X-ray were most frequently reported.

Outcomes measured for identifying low-value examinations varied across the studies and the most common were diagnostic yield (n = 213), and impact or change in treatment or management (n = 137). Importantly, the examinations defined as low-value were dependent on the clinical symptoms of patients e.g. a lumbar spine MRI is only valuable when the patient present with red flag symptoms.

In the following sections, results are stratified by body areas (neurologic (central nervous system [CNS]), thoracic, musculoskeletal, abdominopelvic, vascular, whole body, breast, cardiac, and ear, nose, and throat, and neck imaging) and population type (i.e., adult versus pediatric population).

### Low-value radiological examinations in adults

#### Neurologic imaging

Low-value imaging of the brain was explored in 49 studies [20–66]. Specifically, routine use of head CT or repeat head CT in minor head injury and brain MRI were reported to be low-value for many clinical indications and conditions. The reason for being low-value was either low diagnostic yield where the majority of scans were reported to have no relevant findings, or a low rate in change of management for patients examined. To reduce the use of low-value neurologic imaging the studies indicated that the scan should be warranted based on patient symptoms rather than routine. Details are presented in Table 3.

**Table 3:** Overview of low-value imaging of the head and brain with reported outcome and suggested practice

Type of imaging	Reason for examination	Outcome	Suggested practice by included study/studies	References
Head CT	Minor head injury	2–7.4% relevant findings		[20–27]
	Delirium	3–11% relevant findings		[28, 29]
	Headache	2–8% relevant findings	Examine patients after trauma or when life-threatening conditions are expected only	[30–33]
	Hepatic encephalopathy	4% relevant findings	Examine patients with history of head trauma or focal neurologic findings only	[34]
	Meningitis	12–14% relevant findings		[35]
	Hip fracture (geriatric)	< 1–6% relevant findings		[36, 37]
	Medical patients	4% relevant finings		[38]
	Lamotrigine toxicity	No impact on patient management	The condition is clinically misinterpreted as stroke	[39]
Repeat head CT	Minor head injury	0–6.5% had change in management	Examine patients with neurological decline only	[33, 40–50]
	Traumatic brain injury	5.2–11.4% had change in management		[48]
	Delayed intracranial hemorrhage	1% relevant findings	Do not repeat routinely for patients on anticoagulation treatment	[51, 52]
	Traumatic epidural hematomas	7% relevant findings		[53]
Follow-up head CT	Shunt surgery	2.3% reoperated		[54]
	Chronic subdural hematoma	No change in treatment	Do not routinely do an early post-op CT	[55]
	Anterior skull base surgery	12% relevant findings	Examine patients with neurological decline only	[56]
Brain MRI	Multiple sclerosis patients in the emergency department	27.8% relevant findings		[57]
	Pure ground glass nodular adenocarcinomas	No relevant findings		[58]
Follow-up brain MRI	Macroprolactinoma	1.7% relevant findings		[59]
Head CT/Brain MRI	Syncope	0–3.8% relevant findings		[60, 61]
	Migraine	Not recommended in guidelines	Clinical examination and patient history should be enough to refer patient to a specialist	[62]
Head XR	Shunt malfunction	Did not change patient management	CT should be used instead	[63]
Head CTA	In stroke patients after brain MRI	50% relevant findings	Examine patients with neurological decline only	[64)
Carotid ultrasound	Syncope	2.2–2.8% relevant findings		[65, 66]

*XR* X-ray, *CT* computed tomography, *MRI* magnetic resonance imaging

Further, low-value imaging examinations of the cervical spine was identified in eleven studies [67–77] including routine imaging in trauma and routine follow-up after surgery in patients without symptoms (Table 4).

**Table 4:** Reported imaging of the cervical (c)-spine with low-value to patients

Type of imaging	Reason for examination	Outcome	Suggested practice by included study/studies	References
C-spine CT/MRI	Blunt trauma	Identified no fractures in patients with negative clinical examination	Imaging is only required in patients with positive physical examination	[67–70]
	Near hanging	1.4% relevant findings	Imaging is only required in patients with positive physical examination	[71]
Routine c-spine XR	High-energy trauma	Identified no fractures	XR is only required in patients with positive physical examination	[72]
Follow-up c-spine XR	Radiculopathy due to a herniated intervertebral disc or an osteophyte	No change in patient management	Intra operative verification is sufficient	[73]
	Spine fusion	No change in patient management	XR is only required in patients with positive physical examination	[74, 75]
	Anterior cervical discectomy	No patients were reoperated based on imaging	XR patients with clinical deterioration only	[76]
C-spine flexion/extension XR	Neck pain	After normal CT—no change in patient management		[77]

*XR* X-ray, *CT* computed tomography, *MRI* magnetic resonance imaging

#### Thoracic imaging

Thirty-eight studies reported chest X-rays to be low-value, while four studies reported on low-value use of chest CT [78–117]. Of these, eighteen reported on chest X-rays in follow-up after procedures known to cause pneumothorax, where the X-ray did not change management in patients without symptoms [94–112]. Further, routine chest X-ray was found to not change patient management when used as a pre and post op screening, at hospital admission, in medical check-ups, or in staging of cervical and breast cancer. Repeat chest X-ray in trauma and ICU patients was found to be low-value and clinical symptoms should be used as an indicator to do an X-ray [78–93, 113, 118–120]. In CT, low-value examinations were found in emergency department patients, pleural effusion, and in staging of low-grade breast cancer as the diagnostic yield is low [114–116]. Further, repeat chest CT in Covid-19 patients showing clinical improvement was shown to be of low-value [117]. Details are presented in Table 5.

**Table 5:** Reported low-value thoracic imaging

Type of imaging	Reason for examination	Outcome	Suggested practice by included study/studies	References
Routine chest XR	Pre/post-operative Elective surgery	0–4% change in management	XR is indicated pre-op for cancer, trauma, and cardiac patients	[78–81]
	Post-op soft tissue sarcoma and stage I germ cell cancer	No change in management	Use chest CT instead	[82, 83]
	Staging in breast or cervical cancer	2.8% relevant findings		[84, 85]
	Medical check-up	0.25% change in management		[86]
	At admission to hospital	Up to 4% relevant findings		[87, 88]
	Acute abdominal pain	6% change in management		[89]
	Trauma patients	Marginal effect on management		[90, 91]
	Congenital lung malformations	No change in management		[92]
Repeat chest XR	Trauma patients	19% relevant findings	Use routine repeats only when initial chest XR is abnormal	[93]
Routine follow-up chest XR	After thoracic invasive interventions	< 1–5.6% change in management	XR patients with symptoms of pneumothorax only	[94–112]
	ICU patients	< 8% change in management	Image patients with positive physical examination only	[113]
Chest CT	Pleural effusion	4% relevant findings		[114]
	Emergency department patients	About 20% relevant findings		[115]
	Pre-op staging of breast cancer	1.5% relevant findings	Useful for stage III patients only	[116]
Repeat chest CT	Covid-19	No change in management when patient is clinically improving		[117]

*XR* X-ray, *CT* computed tomography

#### Musculoskeletal imaging

##### Spine and hip or pelvis

The most commonly reported low-value procedures in musculoskeletal imaging was for low back pain [121–130]. Ten studies demonstrated that X-ray, CT and MRI have a low impact on the treatment of patients without red flags, and 58.7% of MRI scans were negative [121–131], imaging for pain in the rest of the spine was also shown as low-value [131]. In addition, change in management were only seen in < 1% of routine post-op X-rays after cervical (c)- or lumbar (l)-spine fusion [132–134]. Another study found that even though 93% of the referrals for lumbar MRI were appropriate according to guidelines, only 13% of the scans showed actionable findings [125]. In cases of pelvic fracture or trauma, routine pelvic X-ray had a low impact on treatment. The same was shown for MRI or CT in pelvic ring fracture [91, 135–137]. In hip fracture and hemiarthroplasty, routine post-op X-ray of the hip was low-value for patients without symptoms [138, 139]. One study showed that MRI is low-value in patients with hip pain when an X-ray is already acquired [140]. Details are presented in Table 6.

**Table 6:** Overview of low-value imaging in the spine, pelvis, and hip

Type of imaging	Reason for examination	Outcome	Suggested practice by included study/studies	References
L-spine XR, CT, MRI	Low back pain	Low rate in change of management MRI: 41.3% relevant findings		[121–130]
Post-op L or C-spine XR	Instrumented single-level degenerative spinal fusions	Does not change treatment of patient	Check with fluoroscopy during surgery	[132]
Post-op L-spine XR	Lumbar fusion	0–1% relevant findings	XR if positive physical examination only	[133, 134]
Spine XR	Acute neck or back pain	0.4% relevant findings		[131]
Pelvic XR	Sever trauma	No change in management		[91]
CT/MRI pelvis	Pelvic ring fracture	No change in management		[135]
Routine Pelvic XR	Pelvic fracture	No change in management in patients with painless straight leg raise	Among awake, alert patients without spinal or lower limb injury, painless straight leg raise can exclude pelvic fractures	[136]
	Trauma	10% change in management	XR if positive physical examination only	[137]
Post-op Hip XR	Hip hemiarthroplasty	No change in management	XR if positive physical examination only	[138]
	Hip fracture	No change in management	XR if positive physical examination only	[139]
MRI Hip	Hip pain	After XR—low impact on treatment		[140]

*XR* X-ray, *CT* computed tomography, *MRI* magnetic resonance imaging

##### Upper and lower limb

The second most common studied musculoskeletal low-value examination was MRI in knee pain without red flags, reported in eight studies [121, 141–147]. In addition, MRI of acute Achilles tendon rupture, X-ray of adjoined joints in ankle fracture, and CT of lower extremities stress fractures were also reported as low-value examinations [148–150]. X-ray of the knee changed management in 0–0.7% of patients after ligament reconstructions, tibia plateau fixation, and partial or total knee arthroplasty [151–156]. In the upper limb, shoulder MRI in patients with shoulder pain or rotator cuff tear had a low impact on treatment [157–159]. X-ray of the shoulder in atraumatic shoulder pain or frozen shoulder had a low impact on clinical management [160, 161]. Further, orthopedic trauma, post-op, or post-splinting X-ray gave little to no change in management [162–169]. MRI of the wrist in ligamentous injury changed the surgical plan in 28% of patients and was thus low-value for many patients [170]. On general use of imaging in the musculoskeletal system, four studies showed that skeletal CT for peri-articular fractures (post-op) [171], and long bone cartilaginous lesions (also MRI) [172] were of low-value. Details are presented in Table 7.

**Table 7:** Overview of low-value imaging in upper and lower limbs

Type of imaging	Reason for examination	Outcome	Suggested practice by included study/studies	References
Shoulder MRI	Shoulder pain	20% relevant findings other imaging modalities could not find	Use XR and US instead	[157, 158]
	Rotator cuff tear	9.8% change in management		[159]
Routine shoulder XR	Frozen shoulder	2.3% relevant findings	XR if positive physical examination only	[161]
	Atraumatic shoulder pain	14.9% change in diagnosis 1.7% change in management		[160]
Post-op shoulder XR	Primary anatomic total shoulder arthroplasty	0–5% relevant findings No change in management		[163]
Post-op humerus XR	Supracondylar humerus fracture	Do not change patient management	XR only unstable fractures	[162]
Wrist MRI	Wrist ligamentous injury	28% change in management		[170]
Follow-up wrist XR	Uncomplicated distal radius fracture	Do not change patient management		[164]
	Distal radius fracture Fixation with a Volar Locking Plate	0–4% change in patient management		[165] [166]
	Distal radius fracture	Do not change patient management		[167]
Upper extremity MRI	Work related complaints	No change in management		[173]
Knee MRI	Knee pain	< 1% change in treatment	Use XR first MRI if locking or surgical history or conservative treatment fails	[121, 141–147]
Post-op knee XR	Anterior cruciate ligament reconstruction	Do not change patient management		[151]
	Partial knee arthroplasty	No change in management		[154, 155]
	Primary total knee replacement	Do not change patient management		[156]
	Medial patellofemoral ligament reconstruction	Do not change patient management	Use intra operative fluoroscopy	[152]
Knee/foot XR of adjacent joints	Ankle fracture	Do not change patient management	Use XR if clinical suspicion of fracture near adjacent joints	[150]
Ankle MRI	Acute Achilles Tendon Ruptures	Imaging generally not indicated in guidelines	Use MRI if equivocal examination findings	[149]
Lower limb imaging	Lower extremity stress fractures	Low diagnostic accuracy of CT, XR, US, and scintigraphy	Use MRI as it has the highest sensitivity and specificity	[148]
Post-op lower limb XR	Tibia plateau fixation	0.7% change in patient management		[153]
XR, CT, MRI, bone scans, FDG-PET	Musculoskeletal Tumors	Do not change patient management	Refer patient to specialist at an early stage	[174]
Post splinting skeletal XR	Fractures	Do not change patient management	Use XR only in displaced fractures manipulated during splinting	[169]
Post-op CT of joints	Peri-articular fractures	< 5% change in management		[171]
CT of joints	Orthopedic trauma (spine, pelvis, lower extremities)	25.3% relevant findings		[168]
Musculoskeletal MRI	Long bone cartilaginous lesions	Advanced imaging was used too often	Refer patients to specialist at an early stage	[172]

*XR* X-ray, *CT* computed tomography, *MRI* magnetic resonance imaging

#### Abdominopelvic imaging

In abdominopelvic imaging, eighteen studies reported imaging with low-value in typical emergency or general medicine conditions [175–191]. X-rays for abdominal pain and upper gastrointestinal imaging (UGI) for reflux resulted in a change in management in only 4% of patients and is often of poor diagnostic quality [187–189]. In acute pancreatitis, < 1.2% of CT and MRI examinations yielded relevant findings [175–178]. Low-value imaging related to surgery or other invasive procedure in the abdomen was reported in seven studies [192–198]. Contrast esophagogram had a low impact on treatment in suspected esophageal perforation, and anastomotic leaks after esophagectomy [195, 197, 198]. In addition, staging of cancer using a different kind of MRI or CT in the abdominal/pelvic area was described as low-value in six studies for various types of cancer [199–203]. In urology, abdominal CT in urolithiasis had a low impact on the treatment of patients with self-limiting episodes or at follow-up [190, 204–206]. Renal ultrasound in new-onset acute kidney injury to screen for hydronephrosis led to changes in management in just 1.8% of patients in one study [207]. In addition, retrograde urethrography in penile fracture had a low impact on treatment in patients without hematuria or urethrorrhagia [208]. An overview of low-value imaging in abdominopelvic imaging is given in Table 8.

**Table 8:** Overview of low-value abdominal imaging

Type of imaging	Reason for examination	Outcome	Suggested practice by included study/studies	References
Abdominal XR	Appendicitis Acute gallbladder disease Acute pancreatitis	Low diagnostic accuracy	US or CT should be used	[180]
	Before UGI	No change in management	Use last image hold in fluoroscopy	[181]
	Constipation	No change in management	Clinical examination is sufficient	[183]
	Abdominal pain	4–12% relevant findings		[187, 189]
Abdominal CT	Urolithiasis	1.8% change in management		[190, 204, 206]
	Complicated gallstone disease	Low diagnostic accuracy	Clinical examination or US is superior to CT	[179]
	Acute appendicitis	Avoid for reducing radiation dose	US should be used first. Only use CT if US is inconclusive	[191]
	Acute pancreatitis	< 1.2% relevant findings		[175–178]
Post-op abdominal CT	Urolithiasis	2.6% relevant findings		[205]
CT pelvis	Gastric cancer	2% change in patient management		[209]
Abdominal MRI	Acute pancreatitis	< 1.2% relevant findings		[175–178]
Abdominal US	After CT – Poly trauma	1.1% relevant findings		[185]
Pre-op Abdominal US	Bariatric surgery	1.2% change in surgical plan		[194]
Abdominopelvic CT/MRI	Uterine cancer	10% relevant results		[199]
	Prostate cancer	1% relevant results		[200, 201]
Liver MRI	Colorectal cancer	After CT – No new findings		[202]
Follow-up adrenals MRI	Adrenal cancer	4% change in surgical plan		[203]
Retrograde urethrography	Penile fracture	No change in management	Use for patients with hematuria or urethrorrhagia	[208]
Renal US	New-onset acute kidney injury—hydronephrosis	1.8% change in management		[207]
Contrast esophagogram	Suspected esophageal perforation	Low diagnostic accuracy	CT is a superior examination	[197]
	Anastomotic leaks after esophagectomy	Low diagnostic accuracy	CT and endoscopy are better examinations	[195, 198]
UGI	Gastroesophageal reflux	4.5% change in management		[188]
Post-op UGI	Swallowing difficulty	Low diagnostic accuracy	CT is a better examination	[193, 196]
	After laparoscopy	No change in management		[192]

*XR* X-ray, *CT* computed tomography, *MRI* magnetic resonance imaging, *US* ultrasound, *UGI* upper gastrointestinal imaging

#### Vascular imaging

The two most reported low-value vascular imaging examinations were CTA of the chest in patients with low risk of pulmonary embolism (7 studies) and ultrasound in patients with low risk for deep venous thrombosis (5 studies). Negative result was demonstrated in 97% of examinations [210–221]. Further, CTA of the abdominal aorta after endovascular aneurysm repair (EVAR) in patients without endoleak 1 month after the EVAR procedure, was identified as low-value [222–224]. Ultrasound was reported to be better as surveillance for EVAR patients as ultrasound increased the negative predictive value to 97.6% [222–224]. In addition, CTA was shown to be of low-value in patients with blunt vertebral artery injuries and vascular injuries of the lower limbs [225, 226]. Details are presented in Table 9.

**Table 9:** Reported vascular imaging with low-value to patients

Type of imaging	Reason for examination	Outcome	Suggested practice by included study/studies	References
Chest CTA	Pulmonary embolism	3% relevant findings		[210–216]
Follow-up abdominal aorta CTA	Post EVAR	3.6% relevant findings	Reduce the number of follow-ups in patients with normal CTA with no endoleak 1 month after EVAR	[222, 223]
			Use doppler US as surveillance unless patient has symptoms or abnormalities on first follow-up	[224]
Spine CTA	Blunt vertebral artery injuries	No relevant findings		[225]
Lower extremity CTA	Lower extremity vascular injuries	40% relevant findings	Use CTA only in patients with high clinical suspicion and absence of hard signs	[226]
Routine Compression US	Deep venous thrombosis in patients with Lower Extremity Cellulitis	8% relevant findings		[217]
Routine lower extremity veins US	Asymptomatic leg in patients with deep venous thrombosis	0–0.8% relevant findings		[218]
	Deep venous thrombosis	No relevant findings	Use a D-dimer test together with a Wells score risk factors as screening	[219]
Post-op lower extremity veins US	Deep venous thrombosis	No relevant findings	US pre-op only	[220]
Four extremity vein duplex US	Deep venous thrombosis	7.5% relevant findings		[221]

*CTA* computed tomography angiography, *US* ultrasound, *EVAR* endovascular aneurysm repair

#### Whole body imaging

Whole body imaging examinations were identified as low-value in trauma and oncology in six studies. Whole body scanning in trauma should be made only when clinically indicated [227–232]. In addition, one study identified CT in soft tissue infections as low-value, with the exception of intra-abdominal abscesses [233]. In oncology, whole body imaging used for staging and follow-up was identified as low-value in 18 studies [58, 234–250]. Details on low-value whole body imaging in oncology is presented in Table 10.

**Table 10:** Overview of identified low-value whole body imaging for staging and follow-up in oncology

Type of imaging	Type of cancer	Outcome	Suggested practice by included study/studies	References
*Cancer staging*				
PET/CT	Endometrial	Low diagnostic accuracy		[244]
	Pure ground glass nodular adenocarcinomas	No additional information		[58]
	Non-colorectal gastrointestinal	11.2% change in patient management		[235]
	Adenocarcinoma Early Esophageal	Low diagnostic accuracy		[237]
CT	Localized Diffuse Large B-cell lymphoma	No new information	CT is unnecessary in combination with PET/CT	[245]
Multiparametric MRI	Prostate (low risk)	No change in management		[234]
Bone scan	Prostate (low risk)	< 1% of bone scans gave relevant information	PET/CT and prostate-specific antigen gives better metastasis detection	[251–253]
	Prostate cancer (radical prostatectomy]	52% change in patient management		[254]
CT and PET/CT	Melanoma	No change in staging based on imaging		[241]
	High-Risk Melanoma	18% change in patient management		[236]
	Pancreatic adenocarcinoma	2% relevant findings		[243]
CT, PET, MRI, bone scan	Breast	0.8% risk of distant metastases 15% clinically relevant findings		[238] [242]
*Follow-up*				
Post treatment CT, PET, MRI, bone scan	Breast	No increased disease detection < 12 months after treatment		[250] [246]
Post treatment PET/CT	Early-Stage, Non-bulky Hodgkin Lymphoma	Low risk of disease recurrence		[239]
	Breast Non-Hodgkin lymphoma Hodgkin disease Colorectal Melanoma Lung	31.6% of inappropriate imaging changed patient management		[247]
Surveillance PET/CT	Esophageal	Does not improve 2-year survival		[240]
	Lung	Does not improve 2-year survival		[240]
Post treatment CT and PET/CT	Diffuse large B-cell lymphoma	1.6–1.8% change in patient management		[248]
	Non-Hodgkin lymphoma	22.1% relevant findings		[249]

*PET* positron emission tomography, *CT* computed tomography, *MRI* magnetic resonance imaging

#### Breast imaging

In breast cancer follow-up, mammography or MRI of the breasts less than 1-year after treatment were described as low-value [255–262]. Follow-up of benign breast tumors with short intervals showed only 0–0.5% identified malignancy in three studies, thus low-value to the majority of patients [260–262]. According to one study [263] on male patients only, 0.9% of breast ultrasound or mammography found malignancy. Details are presented in Table 11.

**Table 11:** Overview of identified low-value breast imaging

Type of imaging	Reason for examination	Outcome	Suggested practice by included study/studies	References
Follow-up mammography, breast US/MRI	Benign breast tumors	0–0.5% identified malignancy No reduction in reoperations		[260–262]
Follow-up mammography/Breast MRI	< 1-year follow-up malign tumor	0.3% of patients needed treatment for malign disease	Follow-up is only required after 12 months	[255–262]
Mammography/breast US	Male breast cancer	0.9% relevant findings		[263]

*US* ultrasound, *MRI*  magnetic resonance imaging

#### Cardiac imaging

Stress imaging such as myocardial perfusion imaging (MPI) and echocardiography were described as low-value in low risk patients, and patients with more than one risk factor for cardiac disease [264, 265]. In patients with infective endocarditis, only 10% of the findings in FDG PET/CT of the heart led to changes in treatment [266]. Routine transthoracic echocardiography in acute ischemic stroke patients had relevant findings in 38% of patients, however only 8.5% of patients had additional work-up [267]. Elective coronary angiography investigating coronary heart disease had relevant findings in 40% of patients in one study [268]. Yet another study found that during coronary angiography left ventriculography is of poor quality [269]. An overview of low-value cardiac imaging is given in Table 12.

**Table 12:** Overview of identified low-value examinations in cardiac imaging

Type of imaging	Reason for examination	Outcome	Suggested practice by included study/studies	References
Stress myocardial perfusion imaging	Cardiac disease	27% relevant findings	Use risk stratification to screen patients	[264, 265]
Stress echocardiography	Cardiac disease	18% relevant findings		[265]
Routine transthoracic echocardiography	Acute ischemic stroke	8.5% change in management		[267]
Elective coronary angiography	Coronary heart disease	40% relevant findings	Use risk stratification to screen patients	[268]
Left ventriculography during angiography	Coronary heart disease	Low diagnostic accuracy	Echocardiography, nuclear scintigraphy, or MRI have better diagnostic results	[269]
PET/CT	Infective endocarditis	10% change in treatment		[266]

*PET* positron emission tomography, *CT* computed tomography, *MRI* magnetic resonance imaging

#### Neck and ear, nose, and throat imaging

Post-operative thyroid cancer ultrasound was found to be low-value as 98% of the scans were negative [270] and the risk for relapse is small [271]. Furthermore, increased use of ultrasound uncovered more benign and low-risk cancers [272, 273]. Radioactive iodine scanning found 17% concordant findings with earlier examinations. Thus, fine needle aspiration should be used in diagnostics instead of imaging [274]. Thyroid ultrasound as follow-up after lobectomy found tumor or recurrence in only 1.5% of patients [275]. According to one study, in patients with secondary hyperparathyroidism routine pre-op Tc-^99^ m-sestamibi scans are unnecessary as nodules are found during surgery [276].

X-ray and CT of the sinuses in acute rhinosinusitis did not change patient management [277].

In patients with facial fractures, X-ray and CT was identified as low-value in five studies [278–282], as imaging did not change the management of the patient. One study introduced the use of ultrasound combined with an X-ray, instead of CT in zygomatic arch and mandibular fractures [281]. Another study described MRI of the face for juvenile ossifying fibroma as low-value [283].

Imaging of templar bones was described as low-value in patients with chronic Eustachian tube dysfunction and pre-op for cochlear implants [284, 285]. Details are presented in Table 13.

**Table 13:** Overview of low-value imaging in Neck and e*ar, nose, and throat imaging*

Type of imaging	Reason for examination	Outcome	Suggested practice by included study/studies	References
Post-op thyroid US	Thyroid cancer	2% relevant findings		[270, 271]
Radioactive iodine scanning	Thyroid cancer	Does not find more than other type of imaging	Use fine needle aspiration diagnostics	[274]
Follow-up thyroid US	After lobectomy	1.5% relevant findings		[275]
Pre-op Tc-99 m-sestamibi	Secondary hyperparathyroidism	Nodules are found during surgery		[276]
Sinus CT/XR	Acute rhinosinusitis	Does not change patient management		[277]
Face CT/XR	Facial fracture	Does not change patient management		[278–282]
Face CT	Zygomatic arch/ mandibular fracture	Using other examinations reduce radiation dose with similar quality	Face US often combined with face XR	[281]
Face MRI	Juvenile ossifying fibroma	Low diagnostic accuracy	Face CT is of better quality	[283]
Pre-op templar bones CT	Cochlear implants	14% relevant findings		[284]
Templar bones CT	Chronic Eustachian tube dysfunction	Does not change patient management		[285]

*XR* X-ray, *CT* computed tomography, *MRI* magnetic resonance imaging, *US* ultrasound

### Low-value imaging examinations in children

The use of low-value imaging in pediatric patients was reported in 62 studies presented in Table 14 [168, 286–345]. The most frequently reported low-value examinations were CT and MRI of the head/brain, CT and X-ray related to trauma, chest X-ray, and musculoskeletal X-rays in fracture follow-up.

**Table 14:** Overview of imaging identified as low-value in pediatrics sorted by body system

Type of imaging	Reason for examination	Outcome	Suggested practice by included study/studies	References
*Neuro imaging*				
Head CT	Minor head injury	33–50% relevant findings		[286–288]
	Shunt-related complications	Few relevant findings	MRI diffusion weighted imaging should be used	[289]
Repeat head CT	Skull fracture	No relevant findings	Repeat only if patient develops symptoms	[290, 291]
	Minor head injury	0–6.6% relevant findings		[292, 293]
Brain MRI/CT	Headache	4–28.8% relevant findings		[294–297]
Post-op head XR	Cochlear implant surgery	Do not change patient management		[298]
C-spine CT/XR	Trauma	Of all included patients 12.8% screened with imaging while 0.2% needed treatment	X-ray would suffice	[299]
*Abdominopelvic imaging*				
Abdominal CT	Liver injury	CT should be avoided to reduce the use of ionizing radiation	Physical examination, FAST and Serum Transaminases should be used as screening	[300]
	Abdominal pain	Did not change patient management		[301]
Repeat abdominal CT	Renal trauma	CT should be avoided to reduce the use of ionizing radiation	US should be used instead	[302]
Abdominal MRI	Appendicitis	Do not change patient management		[303]
Abdominal XR	Children doing UGI	Do not change patient management		[304]
	Idiopathic constipation	Low diagnostic accuracy	Clinical examination would be sufficient	[305, 306]
Rectal US				
Colonic transit study				
Thoracoabdominal XR	Determining the Position of Umbilical Venous Catheters	XR should be avoided to reduce the use of ionizing radiation	Use ultrasound instead	[307]
UGI	Laparoscopic Gastrostomy Tube Placement	Do not change patient management		[308, 309]
	Gastroesophageal reflux (neonates)	Do not change patient management		[310]
Scrotal US	Pediatric Cryptorchidism	Low diagnostic accuracy	Clinical examination would be sufficient	[311, 312]
Tc-99 m MAG3/DMSA scan	Multicystic dysplastic kidney	Avoid for reducing the use of ionizing radiation	Use US instead	[313]
*Whole body imaging*				
Trauma CT	Blunt trauma	18% relevant findings		[314–316]
	Falls	Two-fold increase in use of CT		[317]
	Trauma	No relevant findings in low level injury		[287, 318, 319]
		Do not change patient management		[320, 321]
Follow-up torso CT	Hodgkin’s lymphoma	Do not change patient management		[322]
*Musculo-skeletal imaging*				
Skeletal CT	Orthopedic trauma (spine, pelvis, lower extremities]	20% relevant findings		[168]
Post-op humerus XR	Supracondylar humerus fracture	Do not change patient management		[323]
		Do not change patient management		[324]
		Do not change patient management	Type III fractures—XR within 7–10 days post-op or if clinical symptoms	[325]
Elbow XR	Supracondylar humerus fracture	Do not change patient management		[326]
	Wrist fracture	Do not change patient management	Image only children with symptoms	[327]
Follow-up forearm XR	Forearm fracture	Do not change patient management		[328]
Serial follow-up wrist XR	Distal wrist fracture	Do not change patient management		[329]
Routine XR pelvis	Blunt trauma	Do not change patient management	Clinical examination as screening	[330, 331)
Routine follow-up Hip XR and US	Hip dysplasia	Routine follow-up (genetic risk)—do not change patient management		[332]
		XR after normal ultrasound do change patient management		[333]
Routine follow-up calf XR	Physeal facture of distal tibia	Do not change patient management		[334]
Ankle XR	Sever's disease	Low diagnostic accuracy	Clinical examination should be sufficient	[335]
Follow-up Spine XR	Adolescent idiopathic scoliosis	Do not change patient management	4-month control only should suffice	[336]
		Do not change patient management	X-ray only patients with pain	[337]
*Thoracic imaging*				
Chest CT	Esophageal atresia and tracheoesophageal fistula	Do not change patient management		[338]
Chest XR	Chest tube removal	6.4% relevant finding	X-ray symptomatic children only	[339, 340]
	CVC placement	Do not change patient management		[341]
	Pneumonia	Do not change patient management	Use ultrasound chest instead	[342]
	Bronchiolitis	Do not change patient management		[343]
*Cardiac imaging*				
Echocardiogram	Cardiac disease	11% change in patient management		[344]
	Myelomeningocele	Do not change patient management	Critical condition is clinically identifiable	[345]

*XR *X-ray, *CT* computed tomography, *MRI* magnetic resonance imaging, *US* ultrasound

## The quantity in use of low-value examinations

The proportion of low-value examinations varied greatly in the 103 included studies reporting proportion. Seven studies explored low-value imaging in adults on an overarching level with several medical conditions and modalities, showing an overall rate of low-value imaging of 2–31% [346–352].

### Quantity of low-value imaging in adults

**Fig. 2:**
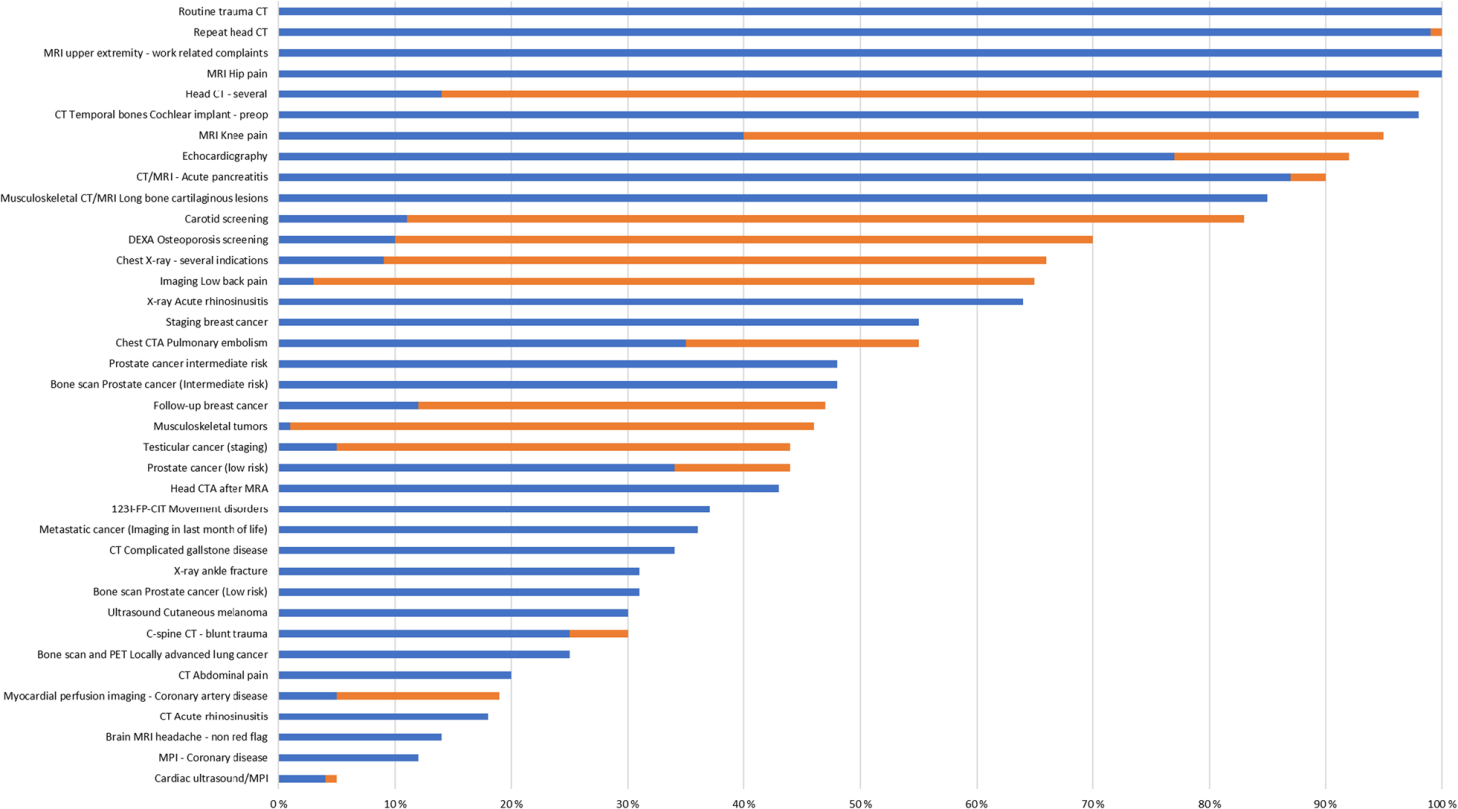
Overview of proportion of low-value examinations in different patient complains/diagnosis. The blue bar represents the minimum rate and the combined blue and orange bar represents the maximum inappropriate rate

The proportion of low-value imaging examinations in specific body areas differed from 4 to 100% (86 studies], and varied both between and within different countries and clinical settings. The results are presented in Fig. 2 [20–23, 26–29, 35, 40–42, 54, 64, 68, 69, 122, 123, 125, 127–130, 140, 141, 143, 145–147, 157–159, 172–174, 176, 177, 179, 182, 190, 210, 211, 213, 215, 216, 229, 242, 252, 277, 284, 353–387]. From these studies, imaging examinations with a high proportion of low-value examinations (more than 50% inappropriate use reported) was: Head CT (routine and repeat), routine trauma scan, MRI in musculoskeletal pain, dual-energy x-ray absorptiometry (DEXA) in low risk patients or low interval DEXA follow-ups, echocardiography, carotid imaging, chest X-ray, X-ray in acute rhinosinusitis, CTA in pulmonary embolism, early-stage breast cancer staging, acute pancreatitis, and special imaging for pre-op templar bone CT in cochlear implantation, and CT/MRI in long bone cartilaginous lesions. In addition, one study reported a sevenfold increase in knee MRI, while there was a reduction in knee arthroscopy [145].

### Quantity of low-value imaging in children

In pediatrics the use of low-value examinations varied between 3.6 and 93.7% (11 studies) [286, 297, 299, 301, 314, 315, 320, 321, 356, 388, 389]. Abdominal CT in appendicitis (3.6%), repeat CT in trauma patients (5%) and C-spine CT in cervical spine injury (13%) were the least over-used examinations. Head CT (50–93.7%), CT scan in case of blunt abdominal trauma (18–80%) and pretransfer CT in trauma patients (66%) were the low-value examinations most used.

## Discussion

In summary, through this scoping review, we found 84 different low-value imaging examinations performed among both adult and pediatric populations, for all imaging modalities, and body areas. Several of these examinations already have established referral criteria or have recommendations against them in the Choosing Wisely list, however this review show that these are still being used in clinical practice, and more examinations might need referral guidelines. The most commonly practices reported as low-value was head CT in several clinical queries (especially related to minor head injury [20–33, 36, 37, 40–53, 55, 56, 286–293]), chest X-ray for routine checkup or follow-ups [78–113, 118–120, 339–343], trauma CT in patients without clinical symptoms or as repeat scans [227–232, 287, 314–322], and skeletal X-rays in non-traumatic pain or in fracture follow-ups [132, 138, 139, 151–156, 160–167, 169, 323–337]. The following were the most frequently reported low-value examinations: imaging in low back pain [121–131] and knee MRI without red flags [121, 141–147], staging and follow-up in several types of cancer (X-ray, CT, MRI and nuclear medicine) [58, 116, 172, 199–203, 209, 234–262], abdominal CT in self-limiting episode of suspected urolithiasis [190, 204–206], chest CTA [210–216] and ultrasound lower limb veins in patients with low risk of thrombosis [217–221] were most prominent among adult populations. When analyzing the extent in use of low-value imaging additional examinations were identified; low interval DEXA screening, echocardiography in patients with low risk of cardiac disease, carotid imaging in syncope, X-ray in rhinosinusitis, and MRI for pain in the hip or upper extremities [140, 157–159, 173, 277, 365, 366, 369, 373, 376].

The variation in the proportion of low-value imaging was large (2–100% inappropriate or unnecessary examinations) and varied between studies of the same examination. There is no obvious threshold in proportion for when to define examinations as low-value. Even though the examinations found in this review are low-value on a group level, certain patient sub-groups or individual patients could have clinical findings justifying the use of imaging. However, in several studies there were identified a rate of ≥ 90% inappropriate imaging examinations. This provides a reason for altering the utilization of these examinations in practice. We found this to be the case in: repeat head or routine trauma CT, echocardiography, MRI in hip, knee and upper extremity pain, CT/MRI in acute pancreatitis, and pre-op templar bone CT in cochlear implantation [40–42, 54, 140, 141, 143, 145–147, 173, 176, 177, 229, 284, 357, 369, 388].

Our review found additional examinations that are potentially low-value to the examinations presented in the Choosing Wisely list [17, 390]. Additionally, we report the extent of low-value imaging. Our additional findings merit further investigation, including chest X-ray after invasive lung procedures such as CVC placement, chest tube placement/removal, biopsies, and other procedures [94–112, 339–341], musculoskeletal follow-ups after fractures or invasive procedures, MRI and X-ray in atraumatic shoulder or upper-extremity pain [138, 139, 151–156, 160–167, 169, 173, 323–329, 334, 336] and staging and follow-up procedures in cancers other than breast, cervical, prostate, and lymphoma [58, 172, 199, 202, 203, 209, 235–237, 239–241, 243–245, 247–249]. Hence, while we confirm previous findings, we also add new findings to the literature. Not all examinations in the Choosing Wisely list were included in this in this review such as cardiac imaging in asymptomatic patients or head CT in patients with sudden hearing loss [390]. This could be caused by the search being incomplete (for instants excluding screening programs), evidence of their low-value was given before 2010 or that some of the Choosing Wisely recommendations were based on clinical experience rather than research reports.

There are many ways to measure low-value imaging, including diagnostic yield, diagnostic accuracy, and impact/change in treatment or management, where diagnostic yield (n = 213) and change in patient management (n = 137) were most common. By applying the Fryback and Thornbury value model as stated by Brady et al. [391], measures of change in patient management and trends in imaging and related treatments, seems a better way to identify low-value imaging, rather than measuring diagnostic accuracy [391].

This scoping review has strengths and limitations in its methods. Although the search in databases was systematic and exhaustive, the cut-off was set at 2010, which excluded examinations identified as low-value imaging or adopted to clinical practice before 2010. Due to the large number of citations retrieved from the database searches, a wide range of inconclusive studies, studies identifying conditional low-value imaging, and articles reporting clinical practice guidelines were excluded. Hence, a wide range of supportive studies were excluded as the inclusion criteria were strict. Therefore, it is likely that there are several studies of low-value examinations that are not included in this review. Accordingly, the excluded studies in Additional file 2 may provide useful information for those who want to pursue specific examinations. The quality of included studies was also not assessed; it is likely that the included studies were of variable quality, limiting the strength of the conclusions made in this review. While the strict inclusion criteria may to some extent compensate for the lack of study quality assessment, quality assessment is not required [392] as the purpose of a scoping review is to identify and map the available evidence. While this review provides a valuable overview of identified low-value imaging, especially useful for clinicians and policymakers to be able to take actions to reduce overuse of diagnostic imaging. However, contextual assessment is needed before changing clinical practice. In addition, the risk of ionizing radiation or contrast media has not been considered in this analysis, this would be interesting issues to consider in later studies. There is also need for research on barriers and facilitators for reducing low-value imaging care to assess where to target policy changes, guidelines, and clinical practice.

## Conclusions

In this study, we provide a comprehensive list of low-value radiological examinations for both adults and children. Our overview reaches beyond earlier published lists and adds information on the quantity of low-value imaging utilization, which reportedly varied from 2 to 100% among included studies. Imaging of atraumatic pain, routine imaging in minor head injury, trauma, thrombosis, urolithiasis, after chest interventions, fracture follow-up and cancer staging, or follow-up were the most frequently identified low-value imaging examinations. This overview can be of great value for clinicians, policymakers, and researchers for revising appropriateness criteria and planning de-implementation. Efforts should be made to reduce the extension and variation of inappropriate imaging which generates huge opportunity costs and is potentially harmful to patients.

## Supplementary Information

**Additional file 1**. Search strategy and hits.

**Additional file 2**. Excluded studies.

**Additional file 3**. Characteristics of the included studies.

## Data Availability

Data sharing is not applicable to this article as no datasets were generated or analyzed during the current study.

## Abbreviations

CT: Computed tomography
CTA: Computed tomography angiography
DEXA: Dual-energy X-ray absorptiometry
EVAR: Endovascular aneurysm repair
MRI: Magnetic resonance imaging
PET: Positron emission tomography
US: Ultrasound
XR: X-ray
