# Supplementary material for: Characterizing and quantifying low-value diagnostic imaging internationally: a scoping review
Source: BMC Med Imaging. 2022 Apr 21;22:73. doi: 10.1186/s12880-022-00798-2 (PMC9022417; doi:10.1186/s12880-022-00798-2)

Additional file 1: Search strategies and hits

**Medline 08. September 2020**

| 1 | diagnostic imaging/ or cardiac imaging techniques/ or imaging, three-dimensional/ or neuroimaging/ or radiography/ or radionuclide imaging/ or respiratory-gated imaging techniques/ or tomography/ or ultrasonography/ or whole body imaging/ |
| --- | --- |
| 2 | exp Radiology/ |
| 3 | (MRI or x-ray* or xray* or ultrasound* or mammography or ultrasonography or DEXA or DXA or CT or radiograph* or radiolog* or tomography or imaging).tw. |
| 4 | (CAT adj scan).tw. |
| 5 | (bone adj scan).tw. |
| 6 | (Magnetic adj resonance adj imaging).tw. |
| 7 | 1 or 2 or 3 or 4 or 5 or 6 |
| 8 | exp Health Services Misuse/ or exp Medical Overuse/ |
| 9 | (Unnecessar* or overuse* or Inappropriate* or waste or wasted or low-value or overdiagn* or overutili* or misuse* or (Low adj value) or unwarrent or redundant).tw. |
| 10 | (Choosing adj wisely).tw. |
| 11 | 8 or 9 or 10 |
| 12 | 7 and 11 |
| 13 | Animal/ not (animal/ and human/) |
| 14 | 12 not 13 |
| 15 | limit 14 to ((danish or Dutch or English or German or Norwegian or Swedish) and last 10 years) |

Results:
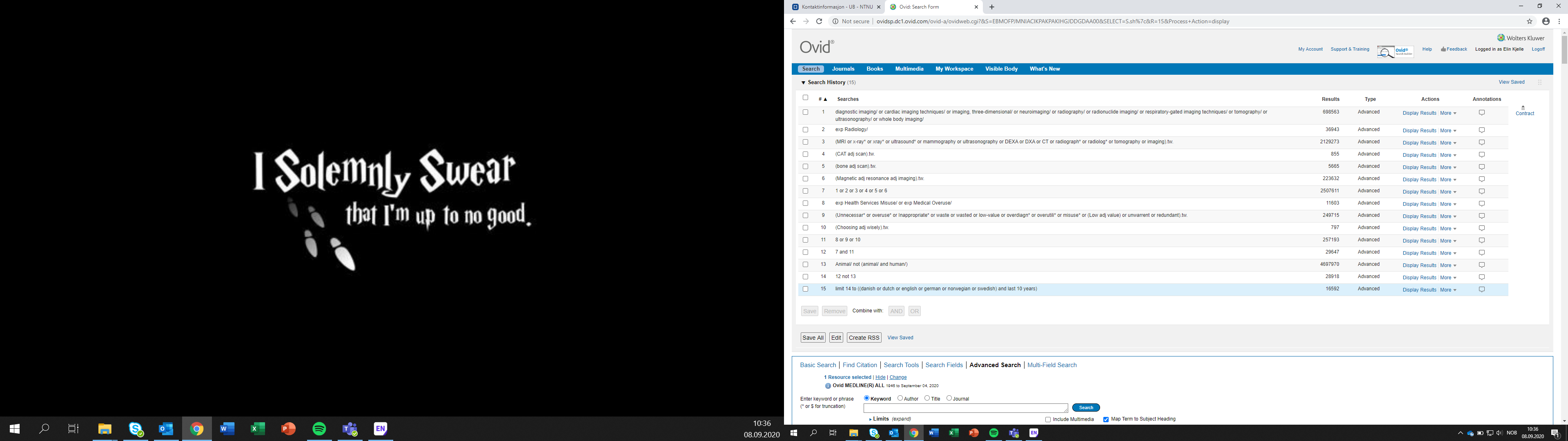


**Scopus 09. September 2020**

Search strategy:

( ( TITLE-ABS-KEY ( "Diagnostic imaging" )  OR  TITLE-ABS-KEY ( radiology )  OR  TITLE-ABS-KEY ( mri )  OR  TITLE-ABS-KEY ( x-ray  OR  xray )  OR  TITLE-ABS-KEY ( ultrasound  OR  ultrasonography )  OR  TITLE-ABS-KEY ( mammography )  OR  TITLE-ABS-KEY ( dexa  OR  dxa )  OR  TITLE-ABS-KEY ( ct )  OR  TITLE-ABS-KEY ( radiograph* )  OR  TITLE-ABS-KEY ( radiolog* )  OR  TITLE-ABS-KEY ( tomography )  OR  TITLE-ABS-KEY ( cat  PRE/  scan )  OR  TITLE-ABS-KEY ( bone  PRE/  scan )  OR  TITLE-ABS-KEY ( "Magnetic resonance imaging" ) ) )  AND  ( ( TITLE-ABS-KEY ( "Health services misuse" )  OR  TITLE-ABS-KEY ( "choosing wisely"  OR  unnecessar*  OR  overuse*  OR  inappropriate*  OR  wasted  OR  low-value  OR  overdiagn*  OR  overutili*  OR  misuse*  OR  unwarrent  OR  redundant ) ) )  AND  ( LIMIT-TO ( SRCTYPE ,  "j" ) )  AND  ( LIMIT-TO ( DOCTYPE ,  "ar" )  OR  LIMIT-TO ( DOCTYPE ,  "re" )  OR  LIMIT-TO ( DOCTYPE ,  "ed" ) )  AND  ( LIMIT-TO ( SUBJAREA ,  "MEDI" )  OR  LIMIT-TO ( SUBJAREA ,  "HEAL" )  OR  LIMIT-TO ( SUBJAREA ,  "MULT" )  OR  LIMIT-TO ( SUBJAREA ,  "ECON" )  OR  LIMIT-TO ( SUBJAREA ,  "NURS" )  OR  LIMIT-TO ( SUBJAREA ,  "SOCI" )  OR  LIMIT-TO ( SUBJAREA ,  "DECI" ) )  AND  ( LIMIT-TO ( PUBYEAR ,  2021 )  OR  LIMIT-TO ( PUBYEAR ,  2020 )  OR  LIMIT-TO ( PUBYEAR ,  2019 )  OR  LIMIT-TO ( PUBYEAR ,  2018 )  OR  LIMIT-TO ( PUBYEAR ,  2017 )  OR  LIMIT-TO ( PUBYEAR ,  2016 )  OR  LIMIT-TO ( PUBYEAR ,  2015 )  OR  LIMIT-TO ( PUBYEAR ,  2014 )  OR  LIMIT-TO ( PUBYEAR ,  2013 )  OR  LIMIT-TO ( PUBYEAR ,  2012 )  OR  LIMIT-TO ( PUBYEAR ,  2011 )  OR  LIMIT-TO ( PUBYEAR ,  2010 ) )  AND  ( LIMIT-TO ( LANGUAGE ,  "English" )  OR  LIMIT-TO ( LANGUAGE ,  "German" ) )  AND  ( EXCLUDE ( EXACTKEYWORD ,  "Human Tissue" )  OR  EXCLUDE ( EXACTKEYWORD ,  "Histopathology" )  OR  EXCLUDE ( EXACTKEYWORD ,  "Unclassified Drug" )  OR  EXCLUDE ( EXACTKEYWORD ,  "Nonhuman" )  OR  EXCLUDE ( EXACTKEYWORD ,  "X Ray Diffraction" )  OR  EXCLUDE ( EXACTKEYWORD ,  "Blood" )  OR  EXCLUDE ( EXACTKEYWORD ,  "Scanning Electron Microscopy" ) OR  EXCLUDE ( EXACTKEYWORD ,  "Immunohistochemistry" )  OR  EXCLUDE ( EXACTKEYWORD ,  "Animals" ) )  AND  ( EXCLUDE ( LANGUAGE ,  "Spanish" )  OR  EXCLUDE ( LANGUAGE ,  "French" )  OR  EXCLUDE ( LANGUAGE ,  "Portuguese" )  OR  EXCLUDE ( LANGUAGE ,  "Italian" )  OR  EXCLUDE ( LANGUAGE ,  "Turkish" )  OR  EXCLUDE ( LANGUAGE ,  "Polish" )  OR  EXCLUDE ( LANGUAGE ,  "Croatian" )  OR  EXCLUDE ( LANGUAGE ,  "Chinese" ) )  AND  ( EXCLUDE ( LANGUAGE ,  "Bulgarian" )  OR  EXCLUDE ( LANGUAGE ,  "Russian" )  OR  EXCLUDE ( LANGUAGE ,  "Serbian" )  OR  EXCLUDE ( LANGUAGE ,  "Persian" )  OR  EXCLUDE ( LANGUAGE ,  "Ukrainian" ) )

Results:


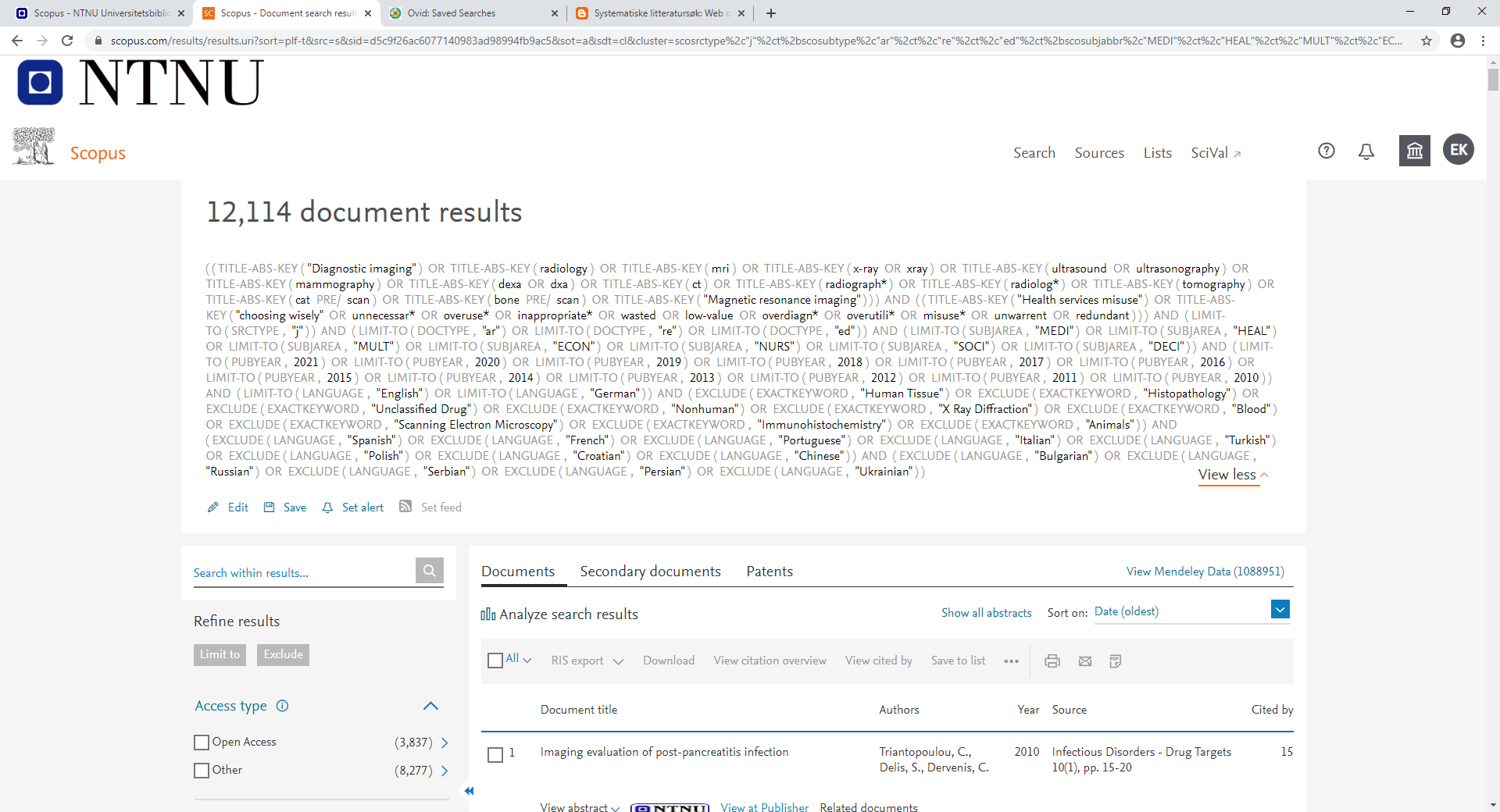


**Cochrane 9. September 2020**

Search strategy

ID Search

#1 MeSH descriptor: [Radiology] explode all trees

#2 MeSH descriptor: [Diagnostic Imaging] explode all trees

#3 MRI

#4 x-ray*

#5 ultrasound*

#6 mammography

#7 ultrasonography

#8 DEXA or DXA

#9 CT

#10 radiograph*

#11 radiolog*

#12 tomography

#13 imaging

#14 {OR #1-#13}

#15 MeSH descriptor: [Health Services Misuse] explode all trees

#16 MeSH descriptor: [Medical Overuse] explode all trees

#17 Unnecessar*

#18 overuse*

#19 Inappropriate*

#20 wasted

#21 "low-value"

#22 overdiagn*

#23 overutili*

#24 misuse*

#25 unwarrent

#26 redundant

#27 "Choosing wisely"

#28 {OR #15-#27} with Cochrane Library publication date Between Jan 2010 and Sep 2020, in Cochrane Reviews

#29 #14 AND #28 with Cochrane Library publication date Between Jan 2010 and Sep 2020, in Cochrane Reviews

Results:


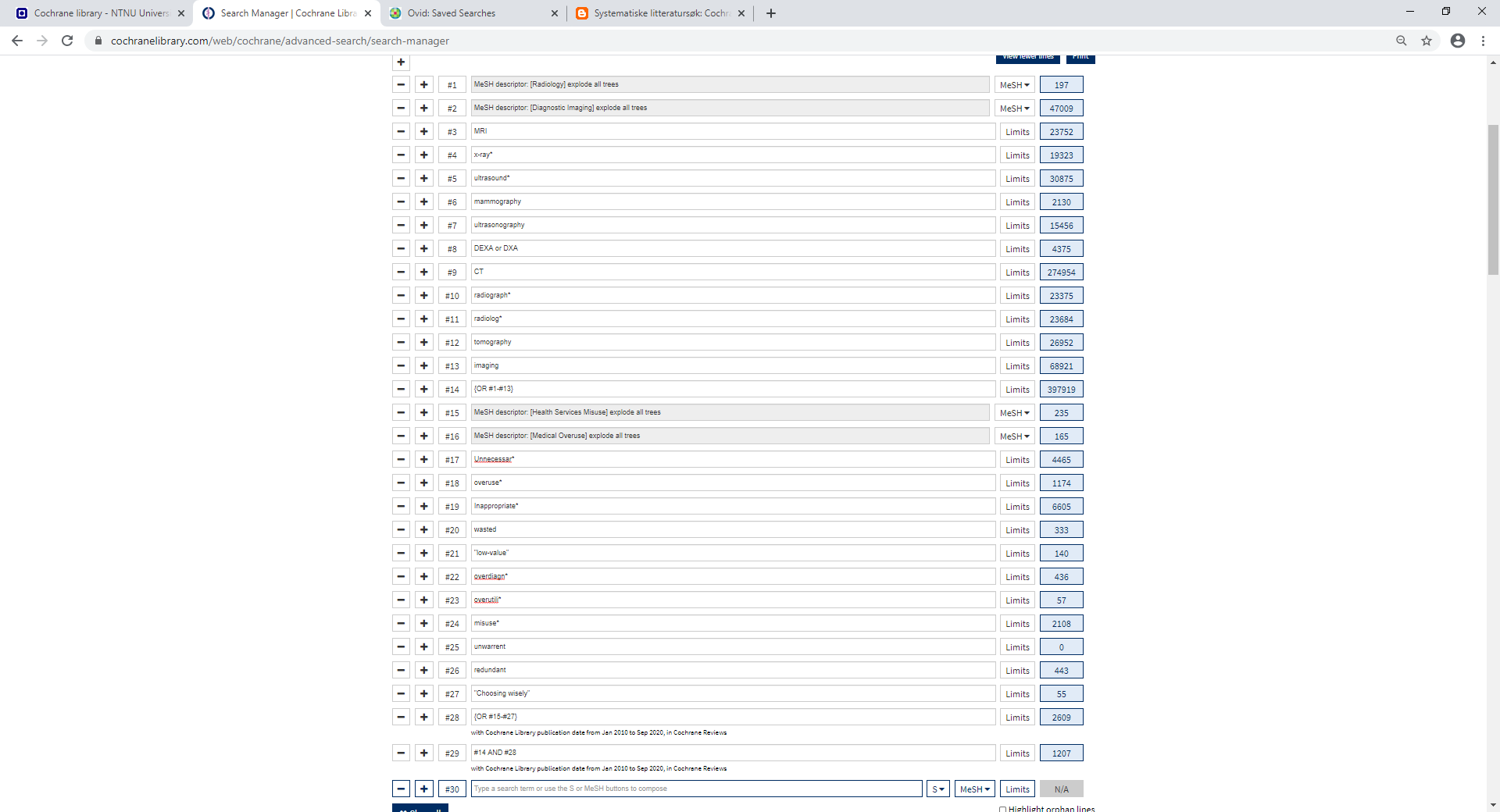


**Embase 11. September 2020**

Search strategy:

| 1. exp diagnostic imaging/ |
| --- |
| 2. exp radiology/ |
| 3. radiolo*.tw. |
| 4. radiograph*.tw. |
| 5. ultraso*.tw. |
| 6. MRI.tw. |
| 7. CT.tw. |
| 8. bone densitometry/ |
| 9. exp mammography/ |
| 10. exp X ray/ |
| 11. exp x-ray computed tomography/ |
| 12. exp nuclear magnetic resonance imaging/ |
| 13. health services misuse.mp. |
| 14. medical overuse.mp. |
| 15. Unnecessar*.tw. |
| 16. overuse*.tw. |
| 17. Inappropriate*.tw. |
| 18. wasted.tw. |
| 19. low-value.tw. |
| 20. overdiagn*.tw. |
| 21. overutili*.tw. |
| 22. misuse*.tw. |
| 23. unwarrent.tw. |
| 24. redundant.tw. |
| 25. "choosing wisely".tw. |
| 26. Animal/ not (animal/ and human/) |
| 27. 1 or 2 or 3 or 4 or 5 or 6 or 7 or 8 or 9 or 10 or 11 or 12 |
| 28. 13 or 14 or 15 or 16 or 17 or 18 or 19 or 20 or 21 or 22 or 23 or 24 or 25 |
| 29. 27 and 28 |
| 30. 29 not 26 |
| 31. limit 30 to (embase and (danish or dutch or english or german or norwegian or swedish) and (article or article in press or editorial or "review") and last 10 years) |

 Results:


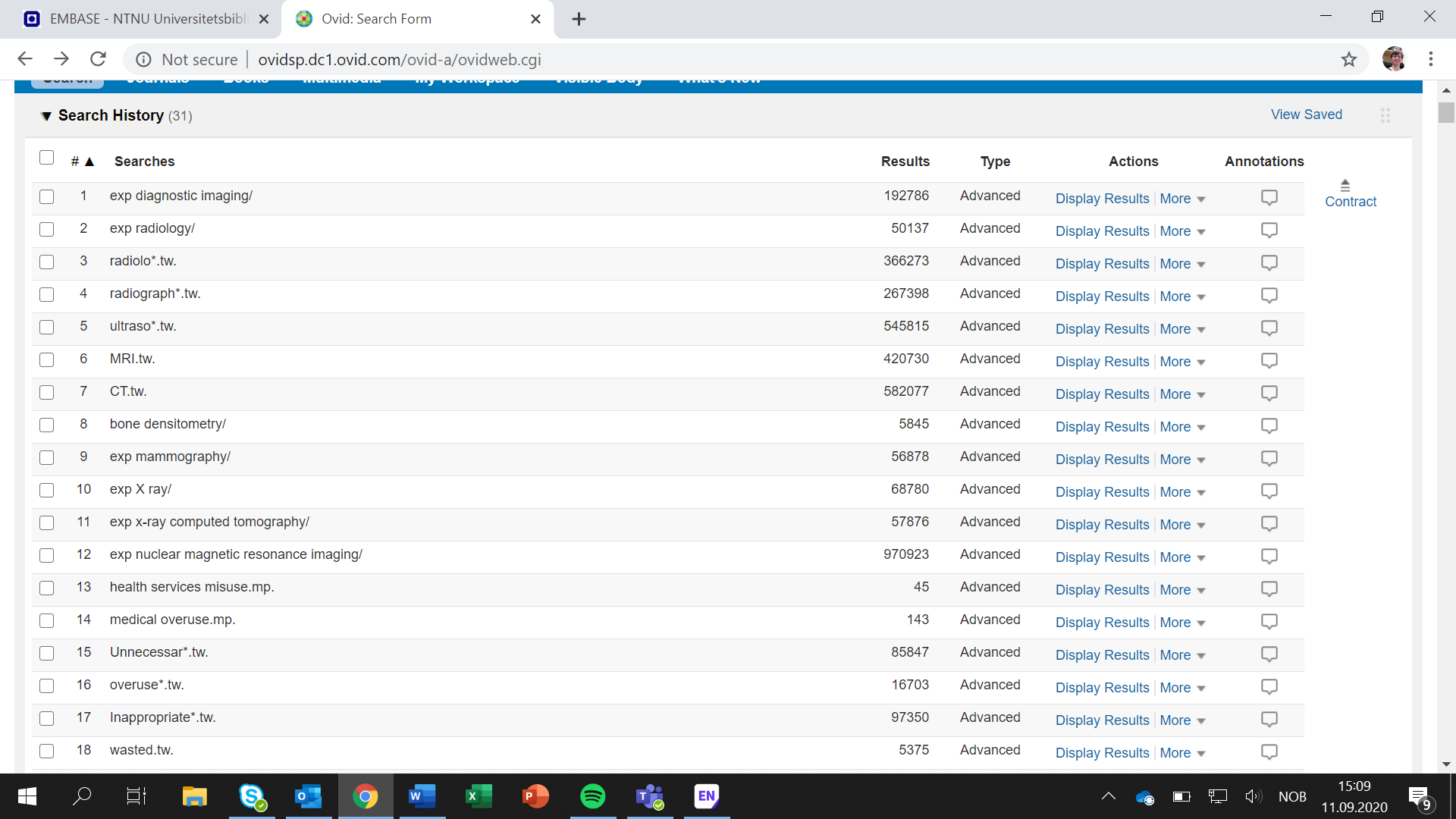


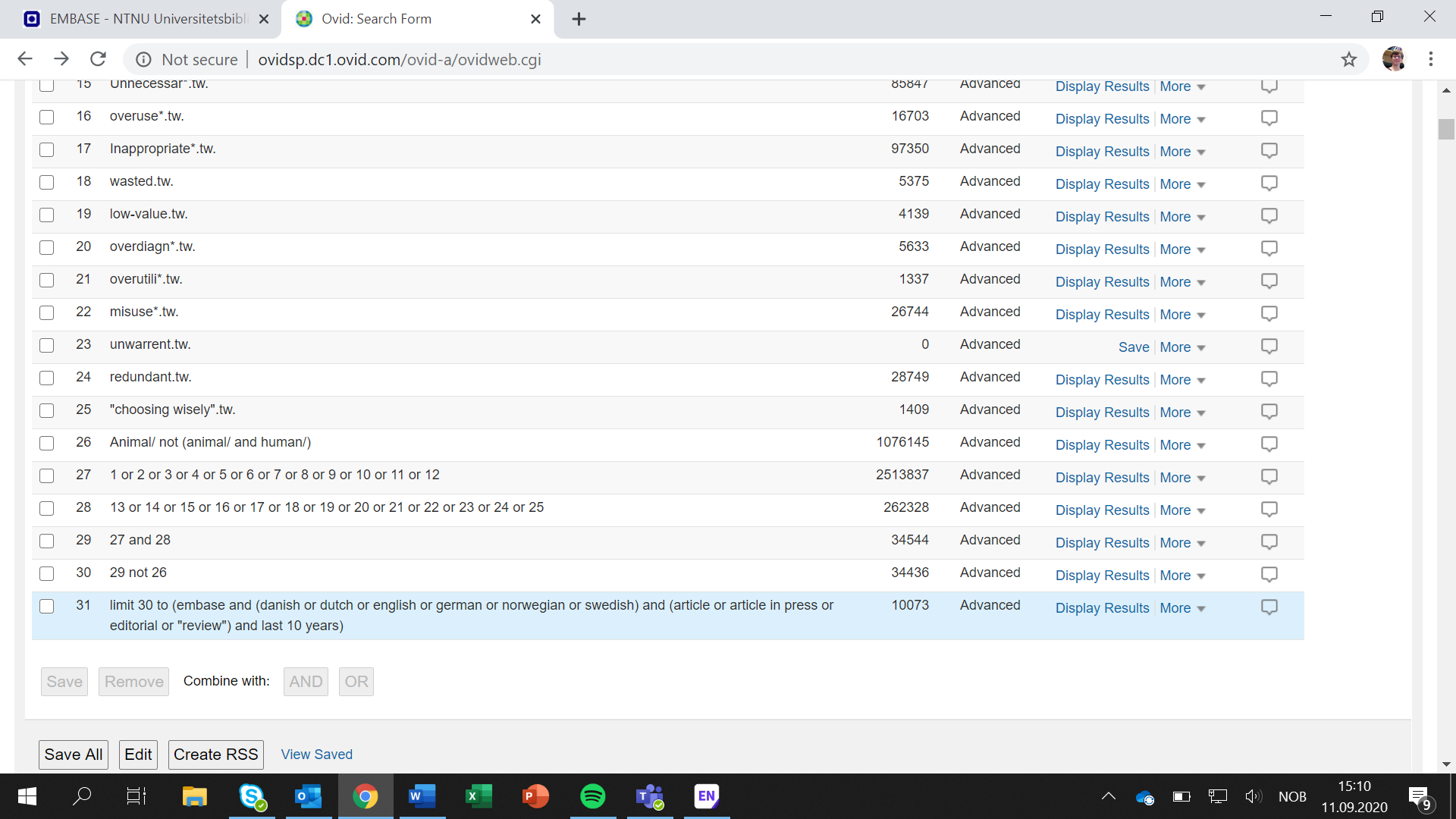

Supplement: Supplementary file 1 — Additional file 1. Search strategy and hits. [file 12880_2022_798_MOESM1_ESM.docx]
