# Supplementary material for: Characterizing and quantifying low-value diagnostic imaging internationally: a scoping review
Source: BMC Med Imaging. 2022 Apr 21;22:73. doi: 10.1186/s12880-022-00798-2 (PMC9022417; doi:10.1186/s12880-022-00798-2)
Supplement: Supplementary file 2 — Additional file 2. Excluded studies. [file 12880_2022_798_MOESM2_ESM.docx]

Additional file 2: Table of papers excluded in full text with reason for exclusion.

| Author | Year | Title | Reason for exclusion |
| --- | --- | --- | --- |
| AANEM | 2015 | AANEM’s top five choosing wisely recommendations. | Guideline |
| Aggarwal et al. | 2010 | Application of the Appropriateness Criteria for Echocardiography in an Academic Medical Center | Wrong study design |
| Aisenberg & Grimes | 2013 | Computed tomography in patients with abdominal pain and diarrhea: does the benefit outweigh the drawbacks? | Not low-value |
| Al Dandan et al. | 2020 | The use of clinical decision rules for pulmonary embolism in the emergency department: a retrospective study | Wrong study design |
| Alhassan et al. | 2016 | Suboptimal implementation of diagnostic algorithms and overuse of computed tomography-pulmonary angiography in patients with suspected pulmonary embolism | Wrong study design |
| Alizadeh et al. | 2011 | Diagnostic Accuracy of Ultrasonic Examination in Suspected Craniosynostosis Among Infants | Wrong study design |
| Al-Jiffry et al. | 2013 | Non-invasive assessment of choledocholithiasis in patients with gallstones and abnormal liver function | Wrong study design |
| Alzhrani et al. | 2019 | Value of Routine Magnetic Resonance Imaging for the Preoperative Assessment of Cochlear Implant Candidates | Not low-value |
| Andersen | 2011 | Is Immediate Imaging Important in Managing Low Back Pain? | Wrong study design |
| Anderson et al. | 2018 | Utility of standardized discharge criteria after appendectomy to identify pediatric patients requiring intervention after postoperative imaging. | Inconclusive |
| Andre et al. | 2011 | Interim FDG-PET Scan in Hodgkin’s Lymphoma: Hopes and Caveats | Wrong study design |
| Archer et al. | 2015 | Regional Cerebral Blood Flow Single Photon Emission Computed Tomography for detection of Frontotemporal dementia in people with suspected dementia (Review) | Wrong outcome |
| Arnold et al. | 2013 | Paediatric blunt abdominal trauma - are we doing too many computed tomography scans? | Inconclusive |
| Arnstead et al. | 2020 | Choosing Wisely Canada rhinology recommendations | Guideline |
| Aro et al. | 2020 | Preoperative evaluation and treatment consideration of parotid gland tumors | Guideline |
| ASCI | 2010 | ASCI 2010 appropriateness criteria for cardiac computed tomography: a report of the Asian Society of Cardiovascular Imaging cardiac computed tomography and cardiac magnetic resonance imaging guideline Working Group | Guideline |
| ASCI | 2010 (2) | ASCI 2010 appropriateness criteria for cardiac magnetic resonance imaging: a report of the Asian Society of Cardiovascular Imaging cardiac computed tomography and cardiac magnetic resonance imaging guideline working group | Guideline |
| Aubry-Bassler et al. | 2013 | Utility of computed tomography and derivation and validation of a score to identify an emergent outcome in 2,315 patients with suspected urinary tract stone | Wrong study design |
| BaRoudsari et al. | 2012 | Burden of Alcohol-Related Injuries on Radiology Services at a Level 1 Trauma Center | Wrong outcome |
| Bartnes et al. | 2011 | Follow-up after acute aortic dissections – time to differentiate? | Wrong outcome |
| Beheshtian et al. | 2020 | Redundant Neurovascular Imaging: Who Is to Blame and What Is the Value? | Wrong outcome |
| Ben Zvi et al. | 2020 | The Utility of Early Postoperative Neuroimaging in Elective/Semielective Craniotomy Patients: A Single-Arm Prospective Trial | Conditional low-value |
| Berezin et al. | 2019 | Lumbosacral spinal imaging for patients presenting to the Emergency department with nontraumatic low back pain | Wrong study design |
| Berretini et al. | 2015 | Bladder urothelial neoplasms in pediatric age: Experience at three tertiary centers | Inconclusive |
| Bhalla et al. | 2015 | Chest tuberculosis: Radiological review and imaging recommendations | Wrong study design |
| Biltman et al. | 2015 | Value of Focused Appendicitis Ultrasound and Alvarado Score in Predicting Appendicitis in Children: Can We Reduce the Use of CT? | Conditional low-value |
| Blozik et al. | 2017 | Medical Overuse in Switzerland: How Frequent is Preoperative Chest Radiography? | Wrong outcome |
| Bokobza et al | 2014 | Pulmonary Embolism Rule-out Criteria vs D-dimer testing in low-risk patients for pulmonary embolism: a retrospective study | Conditional low-value |
| Bot et al | 2017 | Accuracy of Intraoperative Computed Tomography during Deep Brain Stimulation Procedures: Comparison with Postoperative Magnetic Resonance Imaging | Wrong outcome |
| Botelho et al | 2020 | Pediatric trauma primary survey performance among surgical and non-surgical pediatric providers in a Brazilian trauma center | Wrong study design |
| Cain et al | 2018 | Emergency Department Use of Neuroimaging in Children and Adolescents Presenting with Headache | Wrong study design |
| Carmona Echeverria et al. | 2012 | Bone Scan Is of Doubtful Value as a First Staging Test in the Primary Presentation of Prostate Cancer | Conditional low-value |
| Carpenter et al | 2020 | Inappropriate imaging for management of cryptorchidism: Has the choosing Wisely recommendation reduced occurrence? | Wrong study design |
| Chairat et al. | 2013 | Are Both Ultrasonography and Mammography Necessary for Cancer Investigation of Breast Lumps in Resource-Limited Countries? | Not low-value |
| Chen et al | 2015 | The prevalence and impact of defensive medicine in the radiographic workup of the trauma patient: a pilot study | Wrong outcome |
| Cheung et al | 2017 | Are We Choosing Wisely in Lymphoma? Excessive Use of Surveillance CT Imaging in Patients With Diffuse Large B-cell Lymphoma (DLBCL) in Long-term Remission | Wrong study design |
| Cheung et al. | 2019 | Decreasing Overutilization of Echocardiograms and Abdominal Imaging in the Evaluation of Children with Fungemia | Wrong study design |
| Chiappetta et al. | 2018 | Postoperative chest ultrasound findings and effectiveness after thoracic surgery: a pilot study | Wrong study design |
| Chmiel et al. | 2019 | Over-ordering of ultrasound and pre-operative investigations for inguinal hernia repair at Northern Health: a Choosing Wisely audit | Wrong study design |
| Cho et al | 2018 | Diagnosis of Cerebral Aneurysm Via Magnetic Resonance Angiography Screening: Emphasis on Legal Responsibility Increases False Positive Rate | Wrong outcome |
| Cohen et al. | 2019 | Low-Value Diagnostic Imaging Use in the Pediatric Emergency Department in the United States and Canada | Wrong outcome |
| Coon et al. | 2018 | 2017 Update on Pediatric Medical Overuse, A Review | Wrong study design |
| Cooper et al. | 2011 | Positron emission tomography (PET) for assessment of axillary lymph node status in early breast cancer: A systematic review and meta-analysis | Wrong outcome |
| Corpman et al. | 2019 | Posttreatment surveillance PET/CT for HPV-associated oropharyngeal cancer | Wrong outcome |
| Crownover & Bepko | 2013 | Appropriate and Safe Use of Diagnostic Imaging | Wrong study design |
| Cyramska-Chyrek et al | 2017 | Diagnostic pitfalls of adrenal incidentaloma | Wrong study design |
| D’Onforio et al. | 2017 | Unenhanced magnetic resonance imaging immediately after radiofrequency ablation of liver malignancy: preliminary results | Wrong outcome |
| Dai et al. | 2018 | The HEART score is useful to predict cardiovascular risks and reduces unnecessary cardiac imaging in low-risk patients with acute chest pain | Conditional low-value |
| Dayananda et al | 2018 | A selective non-operative approach to thoracic stab wounds is safe and cost effective – a South African experience | Conditional low-value |
| De Lambert et al. | 2016 | Surgical Management of Neuroendocrine Tumors of the Appendix in Children and Adolescents: A Retrospective French Multicenter Study of 114 Cases | Inconclusive |
| De Rubeis | 2020 | Radiological outpatient’ visits to avoid inappropriate cardiac CT examinations: an 8‑year experience report | Conditional low-value |
| Dean Deyle | 2011 | The role of MRI in musculoskeletal practice: a clinical perspective | Wrong study design |
| Deng et al | 2015 | Xanthogranulomatous cholecystitis mimicking gallbladder carcinoma: An analysis of 42 cases | Wrong study design |
| Denjagic et al | 2019 | Justification of radiological procedures algorithm adjustment in diagnosis of lower back pain cause at university clinical center Tuzla | Wrong study design |
| DGAI, DGCH & DGIM | 2017 | Präoperative Evaluation erwachsener Patienten vor elektiven, nicht Herz-Thorax-chirurgischen Eingriffen | Guideline |
| Dhakal et al | 2019 | Overutilisation of imaging studies for diagnosis of pulmonary embolism: are we following the guidelines? | Wrong study design |
| Eisenmenger & Anzai | 2016 | Computed Tomography in Pediatric Traumatic Brain Injury: Who Needs It and How Is It Scored? | Wrong study design |
| Ellison et al | 2018 | Follow-up imaging after acute evaluations for pediatric nephrolithiasis: Trends from a National database | Wrong study design |
| Enright et al. | 2018 | Factors associated with imaging in patients with early breast cancer after initial treatment | Wrong outcome |
| Esparaz et al. | 2019 | A simple algorithm to improve quality while reducing resource utilization in evaluation of suspected appendicitis in children | Guideline |
| Evangelista et al. | 2016 | Hand-held cardiac ultrasound screening performed by family doctors with remote expert support interpretation | Not low-value |
| Ferrari | 2016 | Imaging studies in patients with spinal pain Practice audit evaluation of Choosing Wisely Canada recommendations | Wrong study design |
| Foks et al. | 2018 | External validation of computed tomography decision rules for minor head injury: prospective, multicentre cohort study in the Netherlands | Wrong study design |
| Fontaine et al. | 2011 | Does positron emission tomography scanning improve survival in patients undergoing potentially curative lung resections for non-small-cell lung cancer? | Wrong outcome |
| French et al. | 2019 | Imaging use for low back pain by Ontario primary care clinicians: protocol for a mixed methods study – the Back ON study | Wrong study design |
| Friedman et al. | 2015 | The diagnosis and management of allergic rhinitis: summary of recommendations by the south african allergic rhinitis working group (saarwg) 2015 | Guidelines |
| Gans et al. | 2014 | Richtlijn ‘Diagnostiek acute buikpijn bij volwassenen’ | Wrong study design |
| García de Pereda de Blasa et al. | 2017 | ¿Está indicado realizar una tomografía computarizadatorácica urgente en el traumatismo leve-moderado? | Wrong language |
| Gargan et al. | 2019 | Are we Over-Imaging the Obese Patient with Suspected Pulmonary Embolus in Ireland | Wrong outcome |
| Garreth et al. | 2013 | An Evidence-Based Approach to the Efficient Use of CT-imaging in the Neurosurgical Patient | Not low-value |
| Giannakopoulos et al. | 2017 | Radiological findings and radiation exposure during trauma workup in a cohort of 1124 level 1 trauma patients | Not low-value |
| Giannitto et al. | 2018 | Unindicated multiphase CT scans in non‑traumatic abdominal emergencies for women of eproductive age: a significant source of unnecessary exposure | Wrong outcome |
| Gibbons et al. | 2017 | Use of echocardiography in outpatients with chest pain and normal resting electrocardiograms referred to Mayo Clinic Rochester | Wrong study design |
| Godtman et al. | 2019 | Development and validation of a prediction model for identifying men with intermediate- or high risk prostate cancer for whom bone imaging is unnecessary: a nation-wide population-based study | Wrong study design |
| Gray et al. | 2015 | The diagnosis and management of allergic rhinitis: summary of recommendations by the south african allergic rhinitis Working group (SAARWG) 2015 | Wrong study design |
| Griffith et al. | 2011 | Screening Cervical Spine CT in a Level I Trauma Center: Overutilization? | Wrong study design |
| Guhwe et al. | 2016 | Routine 24-Hour Computed Tomography Brain Scan is not useful in stable patients Post Intravenous Tissue Plasminogen Activator | Wrong study design |
| Guo et al. | 2019 | Feasibility of ultrashort echo time images using full-wave acoustic and thermal modeling for transcranial MRI-guided focused ultrasound (tcMRgFUS) planning | Wrong study design |
| Gupta et al. | 2017 | Choosing wisely: The Canadian Thoracic Society's list of six things that physicians and patients should question | Guideline |
| Gupta et al. | 2016 | Evaluating Imaging Follow-Up Strategies and Costs of Unruptured Intracranial Aneurysms Treated with Endovascular Techniques: A Survey of Academic Neurovascular Centers in the United States | Not low-value |
| Halaweish et al. | 2018 | Compliance with evidence-based guidelines for computed tomography of children with head and abdominal trauma | Wrong study design |
| Hartigan et al. | 2014 | Unnecessary diagnostic imaging: a review of the literature on preoperative imaging for boys with undescended testes | Wrong study design |
| Havrda | 2014 | Pretransfer Imaging Decisions in Rural Trauma Cases | Wrong study design |
| Haydon | 2013 | Head injury: Audit of a clinical guideline to justify head CT | Wrong study design |
| Heetderks-Fong | 2019 | Appropriateness Criteria for Neuroimaging of Adult Headache Patients in the Emergency Department How Are We Doing? | Wrong study design |
| Hekimoglu et al. | 2019 | Comparison of ultrasound and physical examination with computerized tomography in patients with blunt abdominal trauma | Not low-value |
| Henry et al. | 2017 | Variation in the Use of Advanced Imaging at the Time of Breast Cancer Diagnosis in a Statewide Registry | Not low-value |
| Herbert et al. | 2018 | Pediatric blunt cerebrovascular injury: the McGovern screening score | Conditional low-value |
| Hinchliffe et al. | 2013 | Transfer of patients with ruptured abdominal aortic aneurysm from general hospitals to specialist vascular centres: results of a Delphi consensus study | Wrong outcome |
| Hirata et al. | 2019 | 11C-methionine-18F-FDG dual-PET-tracer–based target delineation of malignant glioma: evaluation of its geometrical and clinical features for planning radiation therapy | Inconclusive |
| Hoang et al. | 2015 | Overdiagnosis of Thyroid Cancer: Answers to Five Key Questions | Wrong study design |
| Hochhegger et al. | 2015 | PET/CT imaging in lung cancer: indications and findings | Wrong study design |
| Hol et al. | 2015 | Management of pancreatic cysts: s review of the current guidelines | Guidelines |
| Hom et al. | 2016 | R-SCAN: Imaging for Low Back Pain | Wrong study design |
| Hom et al. | 2016 | R-SCAN: Imaging for Headache | Wrong study design |
| Horne et al. | 2014 | Adolescent Idiopathic Scoliosis: Diagnosis and Management | Wrong study design |
| Horne et al. | 2014 | Adolescent Idiopathic Scoliosis: Diagnosis and Management | Wrong study design |
| Houssami et al. | 2014 | Staging the axilla in women with breast cancer: the utility of preoperative ultrasound-guided needle biopsy | Wrong outcome |
| Huang et al. | 2020 | Apical variant hypertrophic cardiomyopathy “multimodality imaging evaluation” | Wrong study design |
| Huang et al. | 2020 | Apical variant hypertrophic cardiomyopathy “multimodality imaging evaluation” | Wrong study design |
| Hubley et al. | 2020 | Geometric and dosimetric effects of image co-registration workflows for Gamma Knife frameless radiosurgery | Wrong outcome |
| Hui et al. | 2014 | A Quality Improvement Initiative to Reduce Unnecessary Follow-up Imaging for Adnexal Lesions | Wrong study design |
| Huo et al. | 2019 | Comparative Effectiveness of CT- vs. Ultrasound-Guided Percutaneous Radiofrequency Ablation among Medicare Patients 65 Years of Age or Older with Hepatocellular Carcinoma | Guideline |
| Hushner et al. | 2017 | Multimodal imaging for clinical target volume definition in prone whole-breast irradiation: a single institution experience | Wrong study design |
| Inaba et al. | 2011 | Evaluation of multidetector computed tomography for penetrating neck injury: A prospective multicenter study | Wrong outcome |
| Jackson et al. | 2019 | Is Routine Spine MRI Necessary in Skeletally Immature Patients With MHE? Identifying Patients at Risk for Spinal Osteochondromas | Wrong outcome |
| Jafari et al. | 2019 | Classifying patients with lumbar disc herniation and exploring the most effective risk factors for this disease | Not low-value |
| James et al. | 2014 | A systematic review of the need for MRI for the clearance of cervical spine injury in obtunded blunt trauma patients after normal cervical spine CT | Not Low-value |
| Jason-Abel | 2016 | Percutaneous biopsy facilitates modern treatment of renal masses | Wrong study design |
| Jayaram et al. | 2019 | Primary Care Referral for Knee MRI in the United Kingdom: Association With Demography and Subsequent Surgical Intervention | Not Low-value |
| Jha | 2015 | The Road to Overdiagnosis: The Case of Subsegmental Pulmonary Embolism | Wrong study design |
| Johnson et al. | 2015 | Image Wisely and Choosing Wisely: Importance of Adult Body CT Protocol Design for Patient Safety, Exam Quality, and Diagnostic Efficacy | Wrong study design |
| Johnson et al. | 2019 | New ACR Choosing Wisely Recommendations: Judicious Use of Multiphase Abdominal CT Protocols | Wrong study design |
| Jones et al. | 2014 | Perioperative Duplex Ultrasound Following Endothermal Ablation of the Saphenous Vein: Is it Worthless? | Wrong study design |
| Jones et al. | 2018 | Pilomatrixoma: A Comprehensive Review of the Literature | Not low-value |
| Joseph et al. | 2015 | Improving Hospital Quality and Costs in Nonoperative Traumatic Brain Injury The Role of Acute Care Surgeons | Wrong study design |
| Kahn et al. | 2010 | Gastro esophageal reflux: An over investigated entity in neonates and infants | Not low-value |
| Kamat et al. | 2015 | Duplication of radiology imaging studies in the emergency department: what is the cost? | Not low-value |
| Kandasamy et al. | 2011 | MR evaluation of biliary-enteric anastomotic stricture: Does contrast-enhanced T1W MRC provide additional information? | Wrong study design |
| Kanzaria et al. | 2015 | Emergency Physician Perceptions of Medically Unnecessary Advanced Diagnostic Imaging | Wrong outcome |
| Kauffman et al. | 2018 | To Scan or Not to Scan: Overutilization of Computed Tomography for Minor Head Injury at a Pediatric Trauma Center | Wrong study design |
| Kelly et al. | 2019 | The Role of CT Angiography in Evaluating Lower Extremity Trauma: 157 Patient Case Series at a Military Treatment Facility | Not low-value |
| Kemp et al. | 2010 | Investigating meniscal symptoms in patients with knee osteoarthritis—Is MRI an unnecessary investigation? | Poor quality |
| Kerley et al. | 2019 | A systematic review of Appropriate Use Criteria for transthoracic echocardiography: are they relevant outside the United States? | Wrong study design |
| Keveson et al. | 2017 | Adding value to daily chest X-rays in the ICU through education, restricted daily orders and indication-based prompting | Intervention |
| Keveson et al. | 2017 | Adding value to daily chest X-rays in the ICU through education, restricted daily orders and indication-based prompting | Wrong study design |
| Kim et al. | 2012 | The Value of Positron Emission Tomography/Computed Tomography for Evaluating Metastatic Disease in Patients With Pancreatic Cancer | Not low-value |
| Kim et al. | 2017 | Point-of-Care Ultrasound Could Streamline the Emergency Department Workflow of Clinically Nonspecific Intussusception | Wrong study design |
| Kindermann et al. | 2014 | Emergency department variation in utilization and diagnostic yield of advanced radiography in diagnosis of pulmonary embolus | Wrong outcome |
| Klang et al. | 2016 | Overuse of Head CT Examinations for the Investigation of Minor Head Trauma: Analysis of Contributing Factors | Wrong study design |
| Korhonen et al. | 2018 | Quantitative and qualitative analysis of bone flap resorption in patients undergoing cranioplasty after decompressive craniectomy | Not low-value |
| Kostov et al. | 2014 | Diagnostic assessment in anterior cruciate ligament (acl) tears | Wrong study design |
| Kuan et al. | 2019 | Indeterminate liver lesions – a virtual epidemic: a cohort study over 8 years | Wrong outcome |
| Kumamaru et al. | 2019 | Large hospital variation in the utilization of Postprocedural CT to detect pulmonary embolism/ Deep Vein Thrombosis in Patients Undergoing Total Knee or Hip Replacement Surgery: Japanese Nationwide Diagnosis Procedure Combination Database Study | Wrong outcome |
| Lang et al. | 2020 | Magnetic Resonance Imaging in Pelvic Fractures - Part 1: Which Criteria Lead Us to Supplementary MRI Diagnostics? | Inconclusive |
| Langer-Gould et al. | 2013 | The American Academy of Neurology’s Top Five Choosing Wisely recommendations | Wrong study design |
| Lee et al. | 2019 | Diagnostic Role of Renal Doppler Ultrasound and Plasma Renin Activity as Screening Tools for Renovascular Hypertension in Children | Inconclusive |
| Lehosit & Cloud | 2015 | Early parkinsonism: distinguishing idiopathic partionson’s disease from other syndromes | Wrong outcome |
| Leis et al. | 2016 | Choosing Wisely Canada – top five list in infectious diseases: An official position statement of the Association of Medical Microbiology and Infectious Disease (AMMI) Canada | Wrong study design |
| Lenci et al. | 2017 | Dermoid cysts: clinical predictors of complex lesions and surgical complications | Wrong study design |
| Levine et al. | 2013 | Variation in use of all types of computed tomography by emergency physicians | Wrong outcome |
| Lewiecki et al. | 2016 | Best Practices for Dual-Energy X-ray absorptiometry Measurement and Reporting: International Society for Clinical Densitometry Guidance | Not low-value |
| Lin et al. | 2016 | Comprehensive Comparison of Multiple-Detector Computed Tomography and Dynamic Magnetic Resonance Imaging in the Diagnosis of Hepatocellular Carcinoma with Varying Degrees of Fibrosis | Not low-value |
| Liu et al. | 2012 | Post-therapeutic Surveillance Schedule for Oral Cancer: Is There Agreement? | Not low-value |
| Lo & Donaldson | 2013 | Vessel Tortuosity Causing False Positives in Detecting Renal Artery Stenosis on Doppler Ultrasound | Not low-value |
| Mackenzie et al. | 2017 | Determinants of Computed Tomography Head Scan Ordering for Patients with Low- Risk Headache in the Emergency Department | Wrong study design |
| Mafi et al. | 2016 | Comparing Use of Low-Value Health Care Services Among U.S. Advanced Practice Clinicians and Physicians | Wrong outcome |
| Makar et al. | 2020 | Variation in the Utilization of Postoperative Computed Tomography for Patients With Nonsyndromic Craniosynostosis: A National Claims Analysis | Wrong outcome |
| Makarov et al. | 2012 | The Population Level Prevalence and Correlates of Appropriate and Inappropriate Imaging to Stage Incident Prostate Cancer in the Medicare Population | Wrong study design |
| Makarov et al. | 2016 | Appropriateness of Prostate Cancer Imaging among Veterans in a Delivery System without Incentives for Overutilization | Wrong study design |
| Makarov et al. | 2013 | Prostate cancer imaging trends after a nationwide effort to discourage inappropriate prostate cancer imaging | Intervention |
| Maley et al. | 2020 | Low-Value Diagnostic Imaging in the Intensive Care Unit A Teachable Moment | Wrong study design |
| Mangus et al. | 2019 | Repeat radiographic imaging in patients with long bone fractures transferred to a pediatric trauma center | Wrong study design |
| Marasco et al. | 2019 | Diagnostic imaging for acute abdominal pain in an Emergency Department in Italy | Wrong outcome |
| Markar et al. | 2011 | Increased use of pre-operative imaging and laparoscopy has no impact on clinical outcomes in patients undergoing appendicectomy | Wrong outcome |
| Masciocchi et al. | 2012 | Quality review: Fleischner criteria adherence by radiologists in a large community hospital | Wrong outcome |
| Mathias et al. | 2012 | Variations in US Hospital Performance on Imaging-use Measures | Wrong outcome |
| Matsevych et al. | 2018 | Diagnostic laparoscopy or selective non‑operative management for stable patients with penetrating abdominal trauma: What to choose? | Not imaging |
| Matsuura et al. | 2020 | Can plain chest X‑ray replace computed tomography for the follow‑up of children who have undergone the Nuss procedure? | Not low-value |
| Matthew et al. | 2018 | CT-FFR to Guide Coronary Angiography and Intervention | Not low-value |
| Matulevicius et al. | 2013 | Appropriate Use and Clinical Impact of Transthoracic Echocardiography | Wrong study design |
| Maung et al. | 2011 | Routine or Protocol Evaluation of Trauma Patients with Suspected Syncope is Unnecessary | Wrong study design |
| Maurer et al. | 2016 | Current use of PSMA–PET in prostate cancer management | Not low-value |
| Mehraj et al. | 2012 | Importance of clinical assessment in diagnosis of acute appendicitis and its role in decreasing negative appendicectomy rate | Wrong outcome |
| Miles et al. | 2019 | Patterns of Surveillance Advanced Imaging and Serum Tumor Biomarker Testing Following Launch of the Choosing Wisely Initiative | Intervention |
| Milks et al. | 2016 | Imaging of primary pediatric lymphoma of bone | Wrong study design |
| Moeri et al. | 2020 | Appropriateness of physicians’ lumbosacral MRI requests in private and public centers in Tehran, Iran | Wrong study design |
| Mohammad et al. | 2013 | Appropriateness of referrals to a tertiary referral centre for bone mineral density testing | Wrong outcome |
| Mohammadi et al. | 2015 | Appropriateness of physicians’ lumbosacral MRI requests in private and public centers in Tehran, Iran | Wrong outcome |
| Morrisroe et al. | 2018 | EVOLVE: The Australian Rheumatology Association’s ‘top five’ list of investigations and interventions doctors and patients should question | Wrong study design |
| Mullally et al. | 2018 | Value of Patient-Directed Brain Magnetic Resonance Imaging Scan with a Diagnosis of Migraine | Wrong outcome |
| Mwinyogle et al. | 2020 | Use of CT Scans for Abdominal Pain in the ED: Factors in Choice | Wrong study design |
| Müller & Sternitzky | 2017 | Klug-entscheiden-Empfehlungen in der Angiologie | Wrong study design |
| Nagy et al. | 2018 | Same-day Routine Chest-X Ray After Thoracic Surgery is Not Necessary! | Wrong study design |
| Neiner et al. | 2016 | Tongue Blade Bite Test Predicts Mandible Fractures Craniomaxillofac Trauma Reconstruction | Inconclusive |
| Neuner et al. | 2019 | Temporal trends and regional variation in the utilization of low‑value breast cancer care: has the Choosing Wisely campaign made a difference? | Intervention |
| Nikbakhsh et al. | 2010 | Preoperative Medical Evaluation in Elective Surgery versus Standard Criteria | Wrong study design |
| Nishtar et al. | 2019 | Rational use of Computed Tomography Scan head in the Emergency Department of a high-volume tertiary care public sector hospital. | Wrong outcome |
| Ntoulia et al. | 2016 | Failed Intussusception Reduction in Children: Correlation Between Radiologic, Surgical, and Pathologic Findings | Not low-value |
| Padmapriyadarsini et al. | 2013 | On chest Xray for TB in HIV-infected persons | Inconclusive |
| Pakpoor et al. | 2020 | Use of Imaging During Emergency Department Visits for Low Back Pain | Wrong outcome |
| Paltiel et al. | 2020 | Limiting surveillance imaging for patients with lymphoma in remission: a mixed methods study leading to a Choosing Wisely recommendation | Wrong outcome |
| Parmar | 2020 | (F)utility of “routine” postprocedural chest radiograph after hemodialysis catheter (central venous catheter) insertion | Wrong study design |
| Phillips et al. | 2011 | The Role of PET in the Treatment of Occult Disease in Head and Neck Cancer: A Modeling Approach | Not low-value |
| Plodpai et al. | 2014 | The Characteristic Differences of Benign Paroxysmal Positional Vertigo among the Elderly and the Younger Patients: A 10-Year Retrospective Review | Wrong outcome |
| Ponger et al. | 2010 | International survey on the management of skin stigmata and suspected tethered cord | Wrong study design |
| Powell-Doherty et al. | 2017 | Examining the role of follow-up skeletal surveys in non-accidental trauma | Inconclusive |
| Pransky et al. | 2015 | "Geographic variation in early MRI for acute work-related low back pain and associated factors." | Wrong outcome |
| Qiu et al. | 2018 | The incremental clinical value of cardiac hybrid SPECT/CTA imaging in coronary artery disease | Inconclusive |
| Quah et al. | 2011 | Computed tomography to detect accessory spleens before laparoscopic splenectomy: is it necessary? | Wrong study design |
| Rachel et al. | 2011 | Is Radiographic Evaluation Necessary in Children With a Clinical Diagnosis of Calcaneal Apophysitis (Sever Disease)? | Inconclusive |
| Rachwan et al. | 2019 | False-positive stress echocardiograms: Predictors and prognostic relevance | Inconclusive |
| Radic & Cochrane | 2018 | Choosing Wisely Canada: Pediatric Neurosurgery Recommendations. | Guideline |
| Rahimi & Rockey | 2016 | Overuse of head CT in cirrhosis with altered mental status | Inconclusive |
| Raja et al. | 2019 | Impact of a Health Information Technology–Enabled Appropriate Use Criterion on Utilization of Emergency Department CT for Renal Colic | Wrong study design |
| Ravindra et al. | 2016 | A 2D threshold of the condylar–C1 interval to maximize identification of patients at high risk for atlantooccipital dislocation using computed tomography | Wrong outcome |
| Rawle & Pighills | 2018 | Prevalence of unjustified emergency department x-ray examination referrals performed in a regional Queensland hospital: A pilot study | Wrong outcome |
| Rawlins W et al. | 2019 | Computed tomography use patterns for pediatric patients with peritonsillar abscess | Wrong study design |
| Resnick et al. | 2017 | Clinical relevance of the routine daily chest X-Ray in the surgical intensive care unit | Wrong study design |
| Rocha et al. | 2020 | Imaging guidelines for acute pancreatitis: when and when not to image | Wrong study design |
| Rostad et al. | 2018 | Multiphase acquisitions in pediatric abdominal-pelvic CT are a common practice and contribute to unnecessary radiation dose | Wrong outcome |
| Roudsari et al. | 2010 | Trends in the utilization of CT for adolescents admitted to an adult level I trauma center | Inconclusive |
| Rubin et al. | 2013 | Emerging and evolving roles for CT in screening for coronary heart disease | Screening |
| Rush et al. | 2018 | The role of MRI in optimizing injury management | Wrong study design |
| Ryan et l. | 2019 | Evidenced-based radiology? A single-institution review of imaging referral appropriateness including monetary and dose estimates for inappropriate scans | Wrong study design |
| Saltik & Basgul | 2014 | When do We Recommend an EEG and Cranial MRI Evaluation for Autistic Children? | Wrong outcome |
| Sarica et al. | 2015 | Computed tomography findings in geriatric trauma patients who admitted to emergency room (service) | Wrong outcome |
| Sarkissian et al. | 2015 | Postoperative fluid collections after colon resection: the utility of clinical assessment | Wrong outcome |
| Schmajuk et al. | 2018 | Variations in radiographic procedure use for Medicare patients with rheumatoid arthritis | Wrong outcome |
| Schnellinger et al. | 2010 | Are Serial Brain Imaging Scans Required for Children Who Have Suffered Acute Intracranial Injury Secondary to Blunt Head Trauma? | Inconclusive |
| Schok et al. | 2014 | Prospective Evaluation of the Added Value of Imaging within the Dutch National Diagnostic Appendicitis Guideline – Do we Forget our Clinical Eye? | Inconclusive |
| Schubert et al. | 2019 | Postoperative chest radiograph after open reduction internal fixation of clavicle fractures: a necessary practice? | Inconclusive |
| Schwartz et al. | 2014 | Measuring Low-Value Care in Medicare | Wrong study design |
| Schwartz et al. | 2018 | Low-Value Service Use in Provider Organizations | Wrong outcome |
| Schöneberg et al. | 2013 | Special considerations in the interpretation of plain radiographs of the cervical spine in children. A review of the literature | Wrong outcome |
| Segard et al. | 2013 | Changing trends in venous thromboembolism-related imaging in Western Australian teaching hospitals, 2002–2010 | Wrong outcome |
| Sheikh et al . | 2012 | Evaluation of acute cervical spine imaging based on ACR Appropriateness Criteria® | Wrong study design |
| Shoughy & Tabbara | 2019 | Initial misdiagnosis of Vogt-Koyanagi-Harada disease | Not imaging |
| Slobodin et al. | 2012 | Incidental computed tomography sacroiliitis: clinical significance and inappropriateness of the New York radiological grading criteria for the diagnosis | Wrong outcome |
| Srinivas et al. | 2012 | Application of “Less Is More” to Low Back Pain | Wrong study design |
| Stanley et al. | 2014 | Emergency Department Practice Variation in Computed Tomography Use for Children with Minor Blunt Head Trauma | Wrong outcome |
| Stauber et al. | 2016 | Prognosis of Low-Risk Young Women Presenting to the Emergency Department With Chest Pain | Not imaging |
| Stephen et al. | 2019 | Intracranial haemorrhage detected bycerebral computed tomography after falls in hospital acute medical wards | Wrong outcome |
| Stout & Nekhlyudov | 2011 | Early Uptake of Breast Magnetic Resonance Imaging in a Community-Based Medical Practice, 2000–2004 | Wrong outcome |
| Sun et al. | 2012 | Coronary CT angiography: How should physicians use it wisely and when do physicians request it appropriately? | Wrong study design |
| Swartzberg & Goldstein | 2018 | High positive computed tomography yields in the emergency department might not be a positive finding | Wrong outcome |
| Sypes et al. | 2020 | Engaging patients in de-implementation interventions to reduce low-value clinical care: a systematic review and meta-analysis | Wrong outcome |
| Systermans & Devitt | 2013 | Computed tomography in acute abdominal pain: an overused investigation? | Wrong outcome |
| Tahvonen et al. | 2020 | The effect of interventions on appropriate use of lumbar spine radiograph and CT examinations in young adults and children: a three-year follow-up | Wrong study design |
| Tan et al. | 2018 | Are We Over diagnosing Pulmonary Embolism? Yes! Paradigm Shift in Pulmonary Embolism | Wrong study design |
| Tan et al. | 2013 | Alvarado score: a guide to computed tomography utilization in appendicitis | Not low-value |
| Tan et al. | 2015 | Prospective Comparison of the Alvarado Score and CT Scan in the Evaluation of Suspected Appendicitis: A Proposed Algorithm to Guide CT Use | Wrong study design |
| Tas et al. | 2020 | Prospective Comparison of the Alvarado Score and CT Scan in the Evaluation of Suspected Appendicitis: A Proposed Algorithm to Guide CT Use | Wrong study design |
| Taskin et al. | 2018 | Problem-solving breast MRI: useful or a source of new problems? | Conditional low-value |
| Taylor et al. | 2010 | ACCF/SCCT/ACR/AHA/ASE/ASNC/NASCI/SCAI/SCMR 2010 Appropriate Use Criteria for Cardiac Computed Tomography | Guideline |
| Tharmatnam et al. | 2019 | Low-Value Transthoracic Echocardiography, Healthcare Utilization, and Clinical Outcomes in Patients With Coronary Artery Disease | Wrong study design |
| Thestrup et al. | 2019 | Comparison of bi- and multiparametric magnetic resonance imaging to select men for active surveillance | Wrong outcome |
| Thiam et al. | 2015 | Clinical Decision Rules for Paediatric Minor Head Injury: Are CT Scans a Necessary Evil? | Wrong study design |
| Thressa et al. | 2020 | Validation of Appropriate Use Criteria for Coronary Computed Tomographic Angiography for Chest Pain Evaluation in a Tertiary Care Emergency Room | Wrong study design |
| Tonismae et al. | 2020 | Anatomical Survey Versus Fetal Echocardiograms for Diagnosis of Cardiac Defects with a Single Umbilical Artery Cases: A Retrospective Cohort Study and Diagnostic Meta-analysis | Conditional low-value |
| Torabi et al. | 2019 | The effect of pain management in reducing limb and spine radiography in stable traumatic patients admitted to the emergency department | Wrong outcome |
| Torfimovaet al. | 2018 | Imaging of Children With Nontraumatic Headaches | Wrong study design |
| Trevisi et al. | 2018 | What Is the Best Timing of Repeated CT Scan in Mild Head Trauma with an Initially Positive CT Scan? | Not low-value |
| Trooboff et al. | 2018 | Choosing Wisely: Optimizing Routine Workup for the Newly Diagnosed Breast Cancer Patient | Wrong study design |
| Tsujimura et al. | 2020 | Intravascular Ultrasound Imaging During Aortoiliac Stenting: No Impact on Outcomes at 1 Year | Inconclusive |
| Tsze et al. | 2019 | Red flag findings in children with headaches: Prevalence and association with emergency department neuroimaging | Wrong outcome |
| Tufano et al. | 2015 | Incidental Thyroid Nodules and Thyroid Cancer Considerations Before Determining Management | Wrong study design |
| Tufescu | 2016 | Working toward reducing postoperative fracture radiographs: a survey of Canadian surgeons | Wrong study design |
| Tullus | 2011 | AAP recommends reduced imaging after first febrile UTI | Wrong study design |
| Tung et al. | 2018 | Factors Associated with Imaging Overuse in the Emergency Department: A Systematic Review | Wrong outcome |
| Turkmen et al. | 2015 | Determination of radiography requirement in wrist trauma | Inconclusive |
| Tuttle et al. | 2010 | The Impact of Sentinel Lymph Node Biopsy and Magnetic Resonance Imaging on Important Outcomes Among Patients With Ductal Carcinoma In Situ | Not low-value |
| Tyler et al. | 2018 | Interventions to Reduce Over-Utilized Tests and Treatments in Bronchiolitis. | Wrong study design |
| Valasek et al. | 2018 | Decreasing Radiograph Errors in Pediatric | Wrong study design |
| Valls et al. | 2016 | FDG-PET Imaging in Hematological Malignancies | Wrong study design |
| Van Castern-Messiodoro et al. | 2014 | Quantitative ultrasound of the heel as triage test to measure bone mineral density compared with dual energy X-ray absorptiometry in men with prostate cancer commencing with androgen deprivation therapy | Conditional low-value |
| Van Cleve et al. | 2011 | Unnecessary Care for Bronchiolitis Decreases With Increasing Inpatient Prevalence of Bronchiolitis | Wrong outcome |
| Van der Velde et al. | 2011 | Comparing the Diagnostic Performance of 2 Clinical Decision Rules to Rule Out Deep Vein Thrombosis in Primary Care Patients | Wrong study design |
| Van Essen et al. | 2010 | (In)appropriate neurosurgical consultation | Wrong study design |
| Van Gerven et al. | 2019 | Reduction of routine radiographs in the follow‐up of distal radius and ankle fractures: Barriers and facilitators perceived by orthopedic trauma surgeons | Wrong outcome |
| Van Ravesteijn et al. | 2012 | The reassuring value of diagnostic tests: A systematic review | Wrong outcome |
| Van Vugt et al. | 2011 | An evidence based blunt trauma protocol | Wrong study design |
| Vanderby et al. | 2017 | Variations in Magnetic Resonance Imaging Provision and Processes Among Canadian Academic Centres | Wrong outcome |
| Vanderby et al. | 2018 | A Day in the Life of MRI: The Variety and Appropriateness of Exams Being Performed in Canada | Wrong study design |
| Vassileva et al. | 2012 | IAEA Survey of Pediatric CT Practice in 40 Countries in Asia, Europe, Latin America, and Africa: Part 1, Frequency and Appropriateness | Wrong outcome |
| Venkatesh et al. | 2020 | Choosing wisely in emergency medicine: Early results and insights from the ACEP emergency quality network (E-QUAL) | Not low-value |
| Venkatesh et al. | 2012 | Evaluation of Pulmonary Embolism in the Emergency Department and Consistency With a National Quality Measure | Wrong study design |
| Verdoorn et al. | 2016 | Predicting High-Flow Spinal CSF Leaks in Spontaneous Intracranial Hypotension Using a Spinal MRI-Based Algorithm: Have Repeat CT Myelograms Been Reduced? | Wrong study design |
| Verhagen et al. | 2017 | Functional abdominal symptoms in children: the role of the abdominal X-ray and measurement of colon transit time | Wrong study design |
| Vidal-Casariego et al. | 2012 | Accuracy of Ultrasound Elastography in the Diagnosis of Thyroid Cancer in a Low-risk Population | Not low-value |
| Viticchi et al. | 2010 | Instrumental investigations and migraine diagnosis | Wrong outcome |
| Von Ziegler et al. | 2012 | Detection of significant coronary artery stenosis with cardiac dual-source computed tomography angiography in heart transplant recipients. | Not low-value |
| Vongchaiudomchoke & Boonyasirinant | 2016 | Positive Pulmonary Computed Tomography Angiography in Patients with Suspected Acute Pulmonary Embolism: Clinical Prediction Rules, Thromboembolic Risk Factors, and Implications for Appropriate Use | Wrong study design |
| Walker et al. | 2015 | Sonographic Evaluation of Hydronephrosis in the Pediatric Population Is Well-Tempered Sonography Necessary? | Wrong study design |
| Walter et al. | 2015 | Intraoperative Radiography for Evaluation of Surgical Miscounts | Inconclusive |
| Wan et al. | 2018 | Repeat computed tomography scans among inter-facility transferred major trauma patients in Oklahoma, 2009–2015 | Wrong outcome |
| Webster et al. | 2014 | The Cascade of Medical Services and Associated Longitudinal Costs Due to Nonadherent Magnetic Resonance Imaging for Low Back Pain | Wrong study design |
| Weidhase et al. | 2013 | Bedeutung der routinemäßigen Röntgenkontrolle nach perkutaner Dilatationstracheotomie Eine prospektive randomisierte Studie | Inconclusive |
| Weinberg et al. | 2010 | Role of Neuroprotein S-100B in the Diagnostic of Pediatric Mild Brain Injury | Wrong study design |
| Weir et al. | 2010 | Trends in use and yield of chest computed tomography | Inconclusive |
| Welk et al. | 2018 | The Impact of the Choosing Wisely Campaign in Urology | Wrong study design |
| Werren et al. | 2019 | Choosing wisely in cardiology: Five proposals from the Italian Association for Cardiovascular Prevention and Rehabilitation | Guideline |
| Westra et al. | 2015 | The incidental pulmonary nodule in a child Part 2: Commentary and suggestions for clinical management, risk communication and prevention | Wrong study design |
| Weyandt et al. | 2013 | Neuroimaging and ADHD: fMRI, PET, DTI Findings, and Methodological Limitations | Wrong study design |
| Weyh et al. | 2019 | Overutilization of Computed Tomography for Odontogenic Infections | Odonatological |
| Wheeler et al. | 2018 | Low back pain Can we mitigate the inadvertent psycho-behavioural harms of spinal imaging? | Wrong study design |
| Wiener et al. | 2014 | An Official American Thoracic Society/American College of Chest Physicians Policy Statement The Choosing Wisely Top Five List in Adult Pulmonary Medicine | Guideline |
| Willens et al. | 2011 | Appropriateness use criteria for transthoracic echocardiography: Relationship with radiology benefit managers preauthorization determination and comparison of the new (2010) criteria to the original (2007) criteria | Guidelines |
| Wilson et al. | 2016 | Screening for Spinal Dysraphisms in Newborns With Sacral Dimples | Wrong study design |
| Woodhouse | 2017 | Bacterial meningitis and brain abscess | Not low-value |
| Worsham et al. | 2016 | Cuboid-navicular coalition in pediatrics: a systematic review and report | Wrong study design |
| Wyler et al. | 2019 | Sinusitis Update | Guideline |
| Xu et al. | 2019 | Validation and comparison of three newly-released Thyroid Imaging Reporting and Data Systems for cancer risk determination | Wrong outcome |
| Xu et al. | 2013 | Over-prescribing of antibiotics and imaging in the management of uncomplicated URIs in emergency departments | Not imaging |
| Yamout et al. | 2020 | Consensus recommendations for the diagnosis and treatment of multiple sclerosis: 2019 revisions to the MENACTRIMS guidelines | Guideline |
| Yang et al. | 2019 | Development and validation of deep learning algorithms for scoliosis screening using back images | Wrong study design |
| Yao | 2012 | The Ottawa knee rules A useful clinical decision tool. | Wrong study design |
| Yates et al. | 2020 | Defining and measuring imaging appropriateness in low back pain studies: a scoping review | Wrong outcome |
| Yazdany et al. | 2013 | Choosing Wisely: The American College of Rheumatology’s Top 5 List of Things Physicians and Patients Should Question | Guideline |
| Yeo et al. | 2014 | Is routine chest X-ray after surgical and percutaneous tracheostomy necessary in adults: a systemic review of the current literature? | Inconclusive |
| Yoo et al. | 2011 | Limited role of interim PET/CT in patients with diffuse large B-cell lymphoma treated with R-CHOP | Inconclusive |
| Yoon & Wann | 2011 | Evaluation of Acute Chest Pain in the Emergency Department “Triple Rule-Out” Computed Tomography Angiography | Wrong study design |
| Young et al. | 2018 | Neuroimaging utilization and findings in headache outpatients: Significance of red and yellow flags | Wrong study design |
| Zadro et al. | 2019 | Choosing Wisely after a sport and exercise-related injury | Wrong study design |
| Zadro et al. | 2019 | Physiotherapists’ views on the Australian Physiotherapy Association’s Choosing Wisely ecommendations: a content analysis | Wrong outcome |
| Zamani et al. | 2018 | The Relationship between Clinical Findings of Shoulder Joint with Bone Damage of Shoulder Joint in Patients with Isolated Shoulder Blunt Trauma | Wrong study design |
| Zamora et al. | 2014 | Overuse of fluoroscopic gastrostomy studies in a children’s hospital | Inconclusive |
