# Supplementary material for: Characterizing and quantifying low-value diagnostic imaging internationally: a scoping review
Source: BMC Med Imaging. 2022 Apr 21;22:73. doi: 10.1186/s12880-022-00798-2 (PMC9022417; doi:10.1186/s12880-022-00798-2)
Supplement: Supplementary file 3 — Additional file 3. Characteristics of the included studies. [file 12880_2022_798_MOESM3_ESM.docx]

**Additional file 3: Characteristics of included studies**

| **Author (year)** | **Country** | **Methods** | **Population** | **Clinical setting** | **Complaint/diagnosis/procedure** | **Low-value practice** |
| --- | --- | --- | --- | --- | --- | --- |
| Abaluck et al. (2016) | USA | Retrospective chart review | 1.9 million visits | Emergency department | PE | Excessive testing |
| Abbas et al. (2015) | USA | Retrospective chart review | 393 children | Hospital | Gastronomy tube placement | Upper GI fluoroscopy |
| Abboud et al. (2017) | USA | Retrospective chart review | 75 patients | Emergency department | MS | Brain MRI |
| Abdelfattah et al. (2012) | USA | Prospective cohort study | 145 patients | Level 1 trauma center | Minor head injury | Consecutive head CT |
| Abdullah et al. (2018) | USA | Retrospective chart review | 197 children | Hospital | UGI fluoroscopic examination | Abdominal XR |
| Abelhad et al. (2019) | USA | Retrospective chart review | 65 children | Hospital | Coronary artery disease | Myocardial perfusion imaging |
| Abou Hussein et al. (2018) | United Arab Emirates | Retrospective chart review | 937 patients | Hospital | Bariatric surgery | Abdominal US |
| Acker et al. (2016) | USA | Retrospective chart review | 500 children | Hospital | Gastrostomy tube placement | Routine UGI |
| Acosta et al. (2020) | USA | Retrospective chart review | 412 children | Hospital | Supracondylar humerus fractures | Elbow XR |
| Adelani et al. (2016) | USA | Prospective cohort study | 599 patients | Orthopedic practice | Knee pain | Knee MRI |
| Agarwal et al. (2018) | India | Retrospective chart review | 1,056 children | Hospital | Minor head injury | Head CT |
| Ahmadina et al. (2014) | Iran | Retrospective chart review | 116 patients | Hospital | Penile fracture | Retrograde urethrography |
| Ahmed et al. (2017) | UK | Prospective cohort study | 292 children | Hospital | Site locked headaches | Brain imaging |
| Aks et al. (2020) | Israel | Retrospective chart review | 178 patients | Hospital | Breast cancer | PET-CT |
| Al Shakarchi et al. (2016) | UK | Systematic review | 2,317 procedures, 7 studies | Hospital | Carotid artery endarterectomy | Duplex ultrasound |
| Alazzawi et al. (2010) | UK | Retrospective chart review | 334 examinations | Hospital | Acute abdominal pain | Chest XR |
| Alberts et al. (2016) | The Netherlands | Retrospective chart review | 122 patients | Tertiary care | Prostate cancer | Multiparametric MRI |
| Alhassan et al. (2018) | USA | Retrospective chart review | 1,220 patients | Hospital | PE | Chest CTA |
| Ali et al. (2013) | Pakistan | Cross-sectional study | 500 patients | Hospital | Preoperative | Chest XR |
| Ali et al. (2018) | Saudi Arabia | Retrospective chart review | 210 patients | Secondary health‑care facility | Headache | Head CT |
| Aljubran et al. (2019) | Saudi Arabia | Retrospective chart review | 77 patients | Hospital | Gastrointestinal cancers | PET-CT |
| Almenawer et al. (2013) | Canada | Single-Center Series and Meta-analysis | 455 patients | Trauma center | Minor head injury | Consecutive head CT |
| Alsamarah et al. (2017) | USA | Retrospective cohort study | 1,475 cases | Hospital | Coronary artery disease | SPECT Myocardial perfusion imaging |
| Anderson et al. (2020) | USA | Serial cross-sectional study | 809,071 examinations | Hospital | Screening carotid bruit, preoperative evaluation, and syncope | Carotid imaging |
| Anne et al. (2017) | USA | Retrospective chart review | 235 children | Tertiary care | Cochlear implant surgery | Postop XR |
| Antoci et al. (2016) | USA | Retrospective chart review | 1,370 patients | Level 1 trauma center | Ankle fracture | Adjacent joints XR |
| Anwar ul Haq et al. (2020) | USA | Retrospective chart review | 1,381 children | Children’s hospital | Constipation | Abdominal XR |
| Aydın & Fatihoğlu (2020) | Tukey | Retrospective chart review | 205 children | Hospital | Hip dysplasia | Follow-up hip US, XR |
| Babbel & Rayan (2012) | USA | Retrospective chart review | 62 patients | Surgical center | Hand pain | Upper extremity MRI |
| Backhus et al. (2014) | USA | Retrospective chart review | 3,808 patients | Hospital | Locally advanced lung cancer | Bone scan, PET |
| Baek et al. (2017) | South Korea | Retrospective chart review | 800 patients | Hospital | Papillary thyroid microcarcinoma | Thyroid US |
| Baek et al. (2017) | South Korea | Retrospective chart review | 186 patients | Hospital | Follicular thyroid carcinoma | Thyroid US |
| Baker et al. (2015) | USA | Retrospective chart review | 448 patients | Cancer center | Uterine cancer | Preoperative CT or MRI |
| Barron et al. (2016) | USA | Retrospective chart review | 342 patients | Hospital | Breast cancer | Mammogram post treatment |
| Barsky et al. (2014) | USA | Retrospective chart review | 149 patients | Hospital | Melanoma | Preoperative PET/CT |
| Bartels et al. (2010) | The Netherlands | Retrospective chart review | 82 patients | Hospital | Cervical radiculopathy | C-spine XR |
| Befeler et al. (2016) | USA | Retrospective chart review | 185 patients | Level 1 trauma center | Traumatic brain injury | Head CT |
| Behmanesh et al. (2019) | Germany | Retrospective, single-center study | 439 patients | Hospital | Hydrocephalus | Head CT |
| Benarroch-Gampel et al. (2011) | USA | Retrospective chart review | 562 patients | Hospital | Complicated gallstone disease | Abdominal CT |
| Benayoun et al. (2016) | USA | Retrospective cross-sectional study | 3,753 patients | Level 1 emergency department | Spine injury | C-spine CT |
| Benedict et al. (2014) | USA | Retrospective cohort study | 262 children | Pediatric trauma center | Injury | CT scans |
| Berger et al. (2012) | Several | Systematic review | 10 papers | Hospital | Idiopathic Constipation | Abdominal XR, colonic transit time, rectal US |
| Bhangu et al. (2010) | UK | Retrospective chart review | 355 patients | Hospital | Appendicitis, acute gallbladder disease or acute pancreatitis | Abdominal XR |
| Biagi et al. (2018 | Italy | Prospective cohort study | 87 children | Hospital | Pneumonia | Chest XR |
| Biondi et al. (2013) | USA | Retrospective chart review | 399 Children | Medical center | Abdominal pain | Abdominal CT |
| Bjerregaard et al. (2015) | Denmark | Retrospective observational study | 1,097 examinations | Hospital | Video-assisted thoracic surgery | Chest XR |
| Bjurlin et al. (2012) | USA | Retrospective chart review | 214 examinations | Hospital | Percutaneous nephrolithotomy | Chest XR |
| Bolt et al. (2018) | Australia | Prospective cohort study | 328 patients | Hospital | Pelvic fracture | Pelvic XR |
| Bouck et al. (2019) | Canada | Retrospective chart review | 97,740 patients | Hospital | LBP | L-spine XR, CT, MRI |
| Brockmeyer et al. (2012) | USA | Retrospective chart review | 319 patients | Hospital | Bariatric surgery | Upper GI fluoroscopy |
| Campiglio et al. (2017) | Italy | Retrospective chart review | 344 patients | Emergency department | Minor head injury | Consecutive head CT |
| Cellina et al. (2018) | Italy | Retrospective chart review | 493 patients | Emergency department | Minor head injury | Head CT |
| Cerfolio et al. (2011) | USA | Retrospective chart review | 1,037 patients | Hospital | Post pulmonary resection | Chest XR |
| Chaudhry et al. (2012) | USA | Retrospective chart review | 1,321 examinations | Level 1 trauma center | Acute non-displaced or minimally displaced fractures | Post splinting XR |
| Cho et al. (2015) | South Korea | Retrospective chart review | 164 patients | Hospital | Pure GGO nodular lung adenocarcinoma | FDG PET/CT and Head MRI |
| Choi et al. (2011) | USA | Retrospective chart review | 6,444 patients | Hospital | Prostate cancer | CT, MRI, bone scan and abdominal US |
| Chui et al. (2018) | Canada | Population-based Retrospective cohort study | 6,875 patients | Tertiary care | CVC | Chest XR |
| Coco & O’Gurek (2012) | USA | Survey analysis | 136,751 patients | Emergency department | PE, acute myocardial infarction, acute coronary syndrome, heart failure, pneumonia, and pleural effusion | Chest CT |
| Connon et al. (2011) | Australia | Prospective observational study | 651 patients | Level 1 trauma center | Traumatic head injury | Consecutive head CT |
| Cook et al. (2010) | USA | Retrospective chart review | 382 children | Level 1 trauma center | Blunt abdominal trauma | Abdominal CT |
| Cortes et al. (2019) | USA | Prospective cohort study | 51 patients | Hospital | Full-thickness rotator cuff tears or other cuff tendinopathies | Shoulder MRI |
| Creeden et al. (2017) | USA | Retrospective chart review | 723 examinations | Hospital | UGI fluoroscopic examinations | Abdominal XR |
| Cruz et al. (2016) | USA | Retrospective chart review | 79 ICU patients | Intensive care unit | Tracheostomy | Chest XR |
| Cuellar et al. (2014) | USA | Retrospective chart review | 79 patients | Hospital | Esophageal Adenocarcinoma | FDG-PET/CT |
| Cunningham et al. (2014) | USA | Retrospective chart review | 462 patients | Hospital | Pneumothorax | Chest XR |
| Dalton et al. (2016) | USA | Retrospective chart review | 622 patients | Hospital | Pneumothorax | Chest XR |
| Danielson et al. (2019) | USA | Retrospective chart review | 713 patients | Hospital | Hip Fracture | Head CT |
| Dawson et al. (2012) | USA | Retrospective chart review | 507 children | Hospital | Minor head injury | Consecutive head CT |
| De Burlet et al. (2018) | New Zealand | Retrospective chart review | 100 CT scans | Hospital | Abdominal pain | Abdominal CT |
| De La Pena et al. (2017) | UK | Retrospective chart review | 1,447 patients | Hospital | Testicular cancer | Chest XR |
| Debald et al. (2014) | Germany | Retrospective chart review | 742 patients | Hospital | Breast cancer | Chest XR, liver US, and bone scan |
| Delnevo et al. (2012) | Italy | Retrospective chart review | 126 patients | Hospital | Several | Chest XR |
| Dempsey et al. (2017) | USA | Retrospective chart review | 160 patients | Hospital | Primary anatomic total shoulder arthroplasty | Shoulder XR |
| Dewi et al. (2016) | UK | Retrospective chart review | 92 patients | Hospital | Open reduction and internal fixation of mandibular fractures | Facial XR |
| Diaz vico & Elli (2015) | USA | Retrospective chart review | 284 patients | Hospital | Bariatric surgery | UGI examination |
| Downie et al. (2020) | Australia | Systematic review | 45 included studies | Several | LBP | L-spine imaging |
| Drangsholt et al. (2019) | USA | Retrospective cohort | 414 patients | Hospital | Prostate cancer | Staging imaging |
| Driver et al. (2020) | USA | Retrospective chart review | 481 patients | Emergency department | Constipation | Abdominal XR |
| Dyer et al. (2013) | UK | Retrospective chart review | 228 examinations | Hospital | Renal colic | CT KUB |
| Eastley et al. (2012) | UK | Retrospective chart review | 138 patients | Hospital | Distal radius fractures | Wrist XR |
| Eisenberg & Kabbaz (2011) | USA | Retrospective chart review | 400 patients | Hospital | Pneumothorax | Chest XR |
| Eisenmann et al. (2020) | Germany | Prospective cohort study | 115 procedures | Hospital | Pneumothorax | Chest XR |
| El-Maadawy et al. (2015) | United Arab Emirates | Prospective comparative study | 74 neonates | Hospital | Umbilical Venous Catheters | Thoracoabdominal XR |
| Eroukhmanoff et al. (2017) | France | Retrospective multicenter study | 115 patients | Tertiary care | Macroprolactinoma | Follow-up MRI |
| Evans et al. (2012) | USA | Retrospective chart review | 738 patients | Medical center | Soft tissue infections | CT |
| Falchook et al. (2014) | USA | Retrospective chart review | 47,224 patients | Hospital | Prostate cancer | Bone Scan |
| Farach et al. (2015) | USA | Retrospective chart review | 6,041 children | Hospital | Trauma | Consecutive CT |
| Farach et al. (2016) | USA | Retrospective chart review | 84 patients | Hospital | Pectus bar removal | Chest XR |
| Farzan et al. (2018) | Iran | Cross-sectional descriptive study | 130 patients | Emergency department | Pneumothorax | Chest XR |
| Fathioglu et al. (2016) | Turkey | Retrospective chart review | 1,012 patients | Emergency department | Several | Chest CT |
| Feder et al. (2018) | USA | Cross-sectional descriptive study | 343 patients | Tertiary care | Shoulder pain | Shoulder XR |
| Ferorelli et al. (2020) | Italy | Retrospective chart review | 100 requests | Emergency department | Head injuries | Head CT |
| Flaherty et al. (2018) | USA | Retrospective cohort study | 184 patients | Level 1 pediatric trauma center | Traumatic epidural hematomas | Consecutive head CT |
| Foy et al. (2014) | USA | Retrospective chart review | 2,662 patients | Hospital | Diagnostic cardiac catheterization and PCI | Stress echocardiography, SPECT |
| Freeman et al. (2013) | Germany | Retrospective chart review | 100 requests | Hospital | Shoulder complaints | Shoulder MRI |
| Gaither et al. (2018) | USA | Retrospective chart review | 145 patients | Trauma centers | Renal trauma | Abdominal CT and renal US |
| Galvão De Lima et al. (2012) | Brazil | Retrospective chart review | 892 patients | Hospital | Renal transplant candidates | Stress Myocardial perfusion imaging |
| Gamss et al. (2015) | USA | Retrospective chart review | 274 adults | Hospital | Hydronephrosis | Kidney US |
| Gandhi et al. (2015) | Canada | Retrospective chart review | 34 children | Hospital | Headache | Head CT |
| Garg et al. (2015) | USA | Retrospective chart review | 23,964 patients | Multiple institutions | Abdominal aortic aneurysms | Follow-up EVAR |
| Garg et al. (2015) | USA | Retrospective chart review | 227 children | Hospital | Idiopathic scoliosis | Spine XR |
| Garras et al. (2012) | USA | Retrospective chart review | 273 patients | Hospital | Acute Achilles Tendon Ruptures | Ankle MRI |
| Gershengorn et al. (2018) | USA | Retrospective cohort study | 321,093 patients | Hospital | Mechanical ventilation | Chest XR |
| Ghaffarpasand et al. (2011) | Iran | Prospective cross-sectional study | 400 patients | Trauma center | High-energy blunt trauma | C-spine XR |
| Golding et al. (2015) | USA | Retrospective chart review | 129 children | Hospital | Wrist fractures | Elbow XR |
| Gómez-García et al. (2018) | Spain | Retrospective cross-sectional study | 300 patients | Primary care | Knee pain | Knee MRI |
| Goodman et al. (2010) | USA | Retrospective chart review | 175 patients | Hospital | Pneumothorax | Chest XR |
| Greig et al. (2019) | USA | Retrospective chart review | 46 patients | Tertiary care | Congenital lung malformations | Chest XR |
| Grimm et al. (2013) | USA | Retrospective chart review | 383 patients | Hospital | Cervical spine fusion | C-spine XR |
| Gulsen et al. (2014) | Turkey | Retrospective chart review | 508 children | Hospital | Minor head injury | Head CT |
| Gunderson & Chang (2014) | USA | Retrospective cohort study | 183 patients | Medical center | DVT | Compression US lower extremities |
| Gunes et al. (2019) | Turkey | Retrospective chart review | 54 children | Emergency department | Shunt-related complications or follow-ups | Head CT |
| Gupta et al. (2010) | USA | Cross-sectional study | 138 patients | Trauma center | Trauma | Consecutive transfer CT |
| Han et al. (2015) | Korea | Observational study | 863 patients | Hospital | Colorectal cancer | Liver MRI after CT |
| Harris et al. (2020) | USA | Retrospective chart review | 548 patients | Hospital | Acute ischemic stroke | TTE |
| Hartridge- Lambert et al. (2013) | USA | Retrospective chart review | 47 patients | Hospital | Non-bulky Hodgkin Lymphoma | PET |
| Haymart et al. (2019) | USA | Longitudinal study | 2,407,440 patients | Hospital | Thyroid cancer | Thyroid US |
| Healy et al. (2016) | USA | Retrospective chart review | 101,598 patients | Hospital | Lung and Esophageal Cancers | PET |
| Heller et al. (2014) | USA | Retrospective chart review | 132 patients | Level 1 trauma center | Trauma | Standard trauma protocol |
| Hentzen et al. (2015) | USA | Retrospective chart review | 65 children | Hospital | Skull fracture | Consecutive head CT |
| Holle et al. (2020) | Denmark | Retrospective cohort study | 178 patients | Hospital | Infective endocarditis | FDG‑PET/CT |
| Holscher et al. (2013) | USA | Retrospective chart review | 174 patients | Level 1 trauma center | Trauma | Chest CT |
| Holtkamp et al. (2017) | Australia | Retrospective chart review | 143 patients | Hospital | Melanoma | CT and PET/CT |
| Hoogendam et al. (2015) | Netherlands | Cross sectional diagnostic study | 288 patients | Hospital | Cervical cancer | Chest XR |
| Hourmozdi et al. (2016) | USA | Retrospective chart review | 1,322 patients | Emergency department | Pneumothorax | Chest XR |
| Hovgaard et al. (2017) | Denmark | Retrospective chart review | 232 patients | Hospital | Soft tissue Sarcomas | Follow-up |
| Howe et al. (2014) | USA | Retrospective chart review | 120 children | Level 1 pediatric trauma center | Minor Head Injury | Consecutive head CT |
| Hu et al. (2017) | China | Retrospective chart review | 846 patients | Hospital | Esophagectomy | Esophagogram |
| Huang et al. (2019) | Taiwan | Retrospective chart review | 3,534 patients | Level 2 trauma center | Head Trauma | Facial CT |
| Hymas et al. (2012) | USA | Retrospective chart review | 1,079 patients | Hospital | Breast cancer | Routine mammogram |
| Imerci et al. (2013) | Turkey | Retrospective chart review | 1,664 patients (adults/children) | Trauma center | Orthopedic trauma | Spine, extremity, or pelvic CT |
| Issa et al. (2014) | USA | Retrospective chart review | 383 patients | Tertiary care | Hip pain | Hip MRI |
| Izamin & Rizal (2012) | Malaysia | Retrospective chart review | 5246 patients | Public health clinic | Part of routine medical examination | Chest XR |
| Izbicki et al. (2016) | Israel | Prospective cohort study | 201 patients | Hospital | Transbronchial Biopsy | Chest XR |
| Jackson et al. (2011) | Australia | Retrospective chart review | 997 patients | Tertiary emergency department | Mixed indications | Abdominal XR |
| Jaecker et al. (2019) | Germany | Prospective cohort study | 56 patients | Hospital | Medial patellofemoral ligament reconstruction | Knee XR |
| Jahanmehr et al. (2019) | Iran | Retrospective chart review | 614 patients | Hospital | Back pain | L-spine MRI |
| Jaukovic et al. (2011) | Serbia | Retrospective chart review | 155 patients | Hospital | Prostate cancer | Bone scan |
| Jaume et al. (2018) | Spain | Prospective observational study | 1,362 patients | Ear, nose, and throat clinic | Acute rhinosinusitis | XR, CT sinuses |
| Jenkins et al. (2018) | Several | Systematic review and meta-analysis | 33 studies | Several | LBP | Several |
| Jennewine et al. (2019) | USA | Retrospective chart review | 142 patients | Level 1 trauma center | Tibia plateau ORIF | Lower extremity XR |
| Johnson et al. (2014) | USA | Retrospective chart review | 167 patients | Hospital | Syncope | Head imaging |
| Johnson et al. (2017) | USA | Retrospective chart review | 162 patients | Hospital | Pneumothorax | Chest XR |
| Jones et al. (2019) | USA | Retrospective chart review | 72 patients | Hospital | Secondary hyperparathyroidism | Tc-99m-sestamibi |
| Kaen et al. (2010) | Spain | Prospective cohort study | 137 patients | Hospital | Minor head injury | Consecutive Head CT |
| Kamal et al. (2016) | USA | Retrospective chart review | 1,143 cases | Cancer center | Breast cancer | CT, PET, MRI, bone scan. |
| Kamper et al. (2020) | Several | Systematic review | The 26 studies, 194,388 patients | Family practice and ED | LBP | XR, CT, MRI |
| Kanaroglou et al. (2015) | Canada | Retrospective chart review | 101,278 children | Hospital | Cryptorchidism | Scrotal US |
| Karalius et al. (2017) | USA | Retrospective chart review | 572 children | Hospital | Supracondylar humerus fractures | XR humerus |
| Karamitopoulos et al. (2012) | USA | Retrospective chart review | 643 patients | Hospital | Supracondylar humerus fractures | XR humerus |
| Karel et al. (2015) | The Netherlands | Meta-analysis | 11 trails | General practitioners | Knee pain and LBP | Routine imaging |
| Kenway et al. (2016) | UK | Retrospective chart review | 175 patients | Tertiary care | Hearing loss | Preoperative head CT |
| Keyhani et al. (2016) | USA | Retrospective cohort study | 4,127 patients | Hospital | Carotid imaging | Several |
| Khan et al. (2010) | Saudi Arabia | Retrospective descriptive case study | 53 children | Hospital | Gastroesophageal reflux | Barium study |
| Khanduja et al. (2018) | USA | Retrospective chart review | 313 patients | Hospital | LBP | L-spine MRI |
| Kim (2016) | South- Korea | Retrospective chart review | 137 patients | Hospital | Lobectomy | Thyroid US |
| Kim et al. (2011) | Korea | Retrospective chart review | 1,703 patients | Hospital | Breast cancer | Chest CT |
| Kim et al. (2020) | USA | Retrospective chart review | 873 children | Trauma center | C-spine injury | CT, XR |
| Kirk et al. (2019) | USA | Retrospective chart review | 12,269 patients | Hospital | Prostate cancer | Bone scan |
| Kirkham et al. (2015) | Canada | Retrospective cohort study | 1,546,223 patients | Hospital | Preop imaging | Chest XR |
| Kirkpartick et al. (2014) | USA | Retrospective chart review | 91 patients | Hospital | Post EVAR control | Frequent controls |
| Kline et al. (2020) | USA | Retrospective chart review | 97,125 patients | Emergency department | PE | Chest CTA |
| Knudsen et al. (2013) | Denmark | Retrospective study | 932 patients | Hospital | Pectus excavatum | Chest XR |
| Kool et al. (2020) | The Netherlands | Cross-sectional study | 3.5 million inhabitants | General practitioner and hospital | LBP | XR, CT, MRI |
| Kose et al. (2010) | Turkey | Prospective cohort study | 80 children | Hospital | Sever's disease | Ankle XR |
| Kothari et al. (2019) | USA | Retrospective chart review | 405 patients | Emergency department | Acute pancreatitis | Pancreatic CT |
| Kröner et al. (2018) | The Netherlands | Observational study | 294 patients | Hospital | Post-op | Chest XR |
| Kumar et al. (2018) | USA | Retrospective chart review | 67 patients | Tertiary care | Hepatic encephalopathy | Head CT |
| Landry et al. (2011) | Canada | Retrospective chart review | 620 referrals | Family doctor | Several | US |
| Lang et al. (2017) | USA | Retrospective chart review | 727 children | Children’s hospital | Cardiac disease | Echocardiograms |
| Lapid et al. (2015) | The Netherlands | Retrospective chart review | 557 patients | Medical center | Breast cancer | Mammograph, US |
| Lasser et al. (2016) | USA | Retrospective longitudinal analysis | 42,320 patients | Ambulatory care | Osteoporosis screening | DEXA |
| Lavery et al. (2011) | USA | Retrospective chart review | 677 patients | Hospital | Prostate cancer | bone scan, pelvic CT, endorectal MRI |
| Lavingia et al. (2015) | USA | Retrospective chart review | 147 patients | Hospital | Fall | Chest/abdominal/pelvic CT |
| Le Gal et al. (2015) | Canada | Retrospective cohort study | 2,804 patients | Hospital | DVT | Asymptomatic leg US |
| Lechtig et al. (2019) | USA | Retrospective chart review | 583 patients | Hospital | Hip hemiarthroplasty | Hip XR |
| Lehnert & Bree (2010) | USA | Retrospective chart review | 459 examinations | Primary care physicians | Different | CT/MRI in general |
| Leichtle et al. (2015) | Germany | Retrospective chart review | 338 patients | Orthopedic practice | Acute neck and back pain | Spine XR |
| Lemmers et a. (2010) | The Netherlands | Retrospective chart review | 509 patients | Level 1 trauma center | Trauma | Consecutive XR |
| Leschber et al. (2014) | Germany | Randomized controlled trial | 95 patients | Hospital | Video-mediastonoscopy | Chest XR |
| Leventer-Roberst et al. (2020) | Israel | Cross-sectional rolling cohort study | 3,689,869 patients, 35,973 children | Hospital | Appendicitis and pre-admission | Abdominal US/CT and Chest XR |
| Li et al. (2018) | USA | Retrospective chart review | 227 patients | Hospital | Gastric cancer | Pelvic CT |
| Linder et al. (2016) | Germany | Retrospective chart review | 1,559,446 patients | Multicenter | LBP | L-spine imaging |
| Ling & Cleary (2018) | Australia | Retrospective chart review | 68 children | Hospital | Distal radius fracture | Serial XR |
| Liu et al. (2015) | Taiwan | Retrospective cohort study | 236,911 patients | Hospital | Metastatic cancer | CT, MRI, PET, Bone scan. |
| Logan et al. (2019) | Australia | Systematic review and meta-analysis | 6 studies | Primary and emergency care | LBP | L-spine CT/XR |
| Loggers et al. (2017) | The Netherlands | Retrospective chart review | 642 patients | Hospital | Hip fracture | Chest XR |
| Longenecker et al. (2017) | USA | Retrospective chart review | 1,184 patients | Hospital | Partial knee arthroplasty | Knee XR |
| Lou et al. (2015) | USA | Retrospective chart review | 264 patients | Hospital | Adrenalectomy | Secondary imaging |
| Louie et al. (2015) | USA | Retrospective chart review | 1,082 patients | Hospital | Breast cancer | Chest XR |
| Lupichuk et al. (2020) | Canada | Retrospective cohort study | 10,142 patients | Hospital | Breast cancer | CT, MRI, PET, Bone scan |
| Mahalik et al. (2012) | India | Prospective cohort study | 30 neonates | Advanced pediatric center | Esophageal atresia and tracheoesophageal fistula | Chest CT |
| Maldonado et al. (2018) | USA | Retrospective chart review | 207 patients | Hospital | Breast cancer | Mammography |
| Maniar et al. (2015) | USA | Retrospective chart review | 1,192 patients | Hospital | Femur fracture | Head CT |
| Manjoros et al. (2013) | USA | Retrospective chart review | 689 patients | Hospital | Breast cancer | MRI, US, mammogram |
| Makarov et al. (2015) | USA | Retrospective cohort study | 39,617 patients | Hospital | Breast and prostate cancer | Staging imaging |
| Martin et al. (2017) | UK | Retrospective chart review | 222 patients | Hospital | Anterior cervical discectomy | C-spine XR |
| Martins et al. (2020) | Portugal | Retrospective chart review | 1,427 requests | Emergency department | Several | CT and US |
| Mateo & Frankel (2015) | USA | Retrospective chart review | 786 patients | Hospital | Breast cancer | Mammogram |
| Maurer et al. (2012) | Germany | Retrospective chart review | 176 patients | Hospital | Multi trauma | Follow-up abdominal US |
| McCammack et al. (2015) | USA | Retrospective chart review | 144 patients | Hospital | Minor head injury | Consecutive head CT |
| McGrath et al. (2017) | USA | Retrospective chart review | 281 children | Tertiary care | Pneumothorax | Chest XR |
| Miccini et al. (2016) | Italy | Retrospective chart review | 302 patients | Hospital | Pneumothorax | Intraoperative fluoroscopy, Chest XR |
| Michelotti et al. (2018) | USA | Retrospective chart review | 140 patients | Tertiary care | wrist ligamentous injury | Wrist MRI |
| Mikhael et al. (2018) | USA | Retrospective chart review | 594 patients | Hospital | Thyroidectomy | Chest XR |
| Milligan et al. (2020) | USA | Retrospective chart review | 13,809 patients | Hospital | Lung cancer | Head CT |
| Misiura et al. (2018) | USA | Retrospective chart review | 172 children | Level 1 pediatric trauma center | Pelvic injury | Pelvic XR before CT |
| Mittadodla et al. (2013) | USA | Retrospective chart review | 185 patients | Hospital | PE | Chest CTA |
| Mittermair et al. (2014) | Austria | Retrospective chart review | 161 patients | Hospital | Laparoscopic sleeve gastrectomy | UGI study |
| Mohammadi et al. (2019) | Canada | Retrospective descriptive study | 615 patients | Hospital | Benign Thyroid Nodules | Follow-up US |
| Molinari et al. (2012) | USA | Retrospective chart review | 100 cases | Hospital | Spinal fusions | L- or C-spine XR |
| Moore et al. (2013) | USA | Retrospective chart review | 174 children | Level 2 trauma center | Blunt trauma | Head CT |
| Moralidis et al. (2013) | Greece | Prospective appropriateness evaluation | 3,032 patients | Hospital | Coronary artery disease | MPI |
| Morden et al. (2014) | USA | Retrospective chart review | >13 million patients | National | Bone density reduction | DEXA |
| Moussa et al. (2014) | USA | Retrospective chart review | 6,603 patients | Hospital | Total knee arthroplasty | Knee XR |
| Moussavi et al. (2018) | Iran | Randomized controlled trial | 140 patients | Emergency room | Blunt trauma | Chest and abdominopelvic CT |
| Nadimi et al. (2014) | USA | Retrospective chart review | 79 patients | Hospital | Anterior skull base surgery | Routine imaging |
| Nagra et al. (2011) | UK | Retrospective chart review | 158 patients | Hospital | Meningitis | Head CT |
| Nally et al. (2019) | USA | Retrospective chart review | 70 patients | Hospital | Blunt vertebral artery injuries | Neck CTA |
| Natoli et al. (2017) | USA | Retrospective chart review | 87 patients | Level 1 trauma center | Pelvic ring injuries | Pelvic CT/MRI |
| Ng et al. (2014) | Singapore | Retrospective chart review | 123 patients | Hospital | Chronic subdural hematoma | Head CT early |
| Nojkov et al. (2013) | USA | Retrospective chart review | 200 patients | Emergency room | Abdominal pain | Repeat abdominal CT |
| Novick et al. (2018) | USA | Retrospective chart review | 241 patients | Hospital | Blunt trauma | C-spine MRI |
| Nystrom et al. (2015) | USA | Prospective consecutive cohort study | 298 patients | Primary care | Musculoskeletal Tumors | XR, CT, MRI, bone scans, and FDG-PET |
| O`Sullivan et al. (2018) | Several | Systematic review | 63 studies, 357,171 patients | Primary care | Several | Several |
| Oikarinen et al. (2013) | Finland | Retrospective chart review | 150 MRI referrals | Hospital | Several | MRI upper abdomen or liver, L-spine, knee, head. |
| Osman et al. (2018) | USA | Retrospective chart review | 295 patients | Emergency department | PE | Chest CTA |
| Owlia et al. (2014) | USA | Retrospective chart review | 130 patients | Hospital | Several | Head CT |
| Owosho et al. (2015) | USA | Retrospective chart review | 6 patients | Hospital | Juvenile ossifying fibroma | MRI |
| Panneerselvan et al. (2013) | USA | Retrospective chart review | 109 patients | Hospital | Thyroid nodules | Radioactive iodine scanning |
| Pappas et al. (2014) | USA | Retrospective chart review | 247 patients | Hospital | Pancreatic adenocarcinoma | Chest CT, PET |
| Parent et al. (2018) | Canada | Retrospective chart review | 215 patients | Hospital | Knee pain | Knee MRI |
| Park et al. (2017) | South Korea | Retrospective chart review | 362 patients | Hospital | Endometrial cancer | FDG-PET/CT |
| Parma et al. (2014) | USA | Retrospective chart review | 438 patients | Level 1 trauma center | Minor head injury | Head CT |
| Patel & Carpenter (2010) | USA | Retrospective chart review | 345 patients | Hospital | Post-EVAR | Abdominal aortic CTA |
| Patel et al. (2010) | USA | Retrospective chart review | 398,978 patients | Hospital | Coronary artery disease | Elective Coronary Angiography |
| Pathak & Parmar (2017) | Canada | Retrospective chart review | 94 patients | Hospital | Pleural effusion | Chest CT |
| Paydar et al. (2012) | Iran | Prospective cross-sectional study | 1,008 patients | Trauma center | Blunt trauma | Routine Chest XR |
| Peck et al. (2011) | USA | Retrospective chart review | 500 patients | Level 1 trauma center | Delayed intracranial hemorrhage | Consecutive head CT |
| Perera et al. (2017) | Australia | Retrospective chart review | 344 patients | Tertiary care | PE | CTA of PE |
| Peres et al. (2019) | Brazil | Cross-sectional study | 465 patients | Primary/secondary care | Migraine | Head CT, MRI |
| Pérez-Cajaraville (2015) | Spain | Multicenter observational study | 6,436 patients | Hospital | LBP | XR, CT, MRI, bone scan |
| Peterson et al. (2014) | USA | Retrospective chart review | 1,848 patients | Hospital | Coronary disease | Stress MPI or echocardiography, coronary angiography, bypass grafting |
| Porter et al. (2020) | USA | Retrospective cohort study | 241 patients | Hospital | Pneumothorax | Chest XR |
| Prasad et al. (2012) | USA | Population based observational cohort study | 30,183 patients | Hospital | Prostate Cancer | CT, bone scan |
| Protack et al. (2018) | USA | Retrospective chart review | 46 patients | Hospital | Vascular injuries | Lower extremity CTA |
| Raffaele et al. (2020) | Italy | Retrospective chart review | 622 children | Hospital | Pneumothorax | Chest XR |
| Rahiminejad et al. (2014) | UK | Retrospective audit and reaudit | 816 patients | Hospital | DVT | Lower limb US |
| Ramey et al. (2020) | USA | Retrospective chart review | 13 patients | Emergency department | Lamotrigine toxicity | Head CT |
| Raza et al. (2017) | USA | Retrospective chart review | 195 patients | Hospital | Stroke | Head CTA after MRI |
| Refahi et al. (2016) | Iran | Survey | 115 patients | Hospital | Knee pain | Knee MRI |
| Reljic et al. (2014) | USA | Systematic review | 10,501 patients, 41 studies | Hospital | Traumatic brain injury | Consecutive head CT |
| Remfry et al. (2015) | Canada | Retrospective chart review | 553 tests | Medical center | Cardiac disease | TEE, SPECT, cardiac catheterization |
| Reynolds et al. (2018) | USA | Retrospective chart review | 166 patients | Tertiary care | Acute pancreatitis | Abdominopelvic CT |
| Rho et al. (2011) | Korea | Retrospective chart review | 1,562 children | Tertiary care | Headache | Head CT, MRI |
| Roberts et al. (2019) | UK | Retrospective chart review | 350 patients | Hospital | Frozen shoulder | Shoulder XR |
| Rodriguez et al. (2018) | USA | Retrospective chart review | 689 patients | Hospital | Blunt trauma | Abdominopelvic CT |
| Rooke & Phillips (2015) | New Zealand | Retrospective chart review | 148 children | Hospital | Forearm fracture | Forearm XR |
| Rose et al. (2012) | USA | Retrospective chart review | 761 patients | Level 1 trauma center | Blunt trauma | C-spine CT |
| Rosen et al. (2018) | USA | Retrospective chart review | 85 patients | Trauma center | Minor head injury | Consecutive head CT |
| Rosenlund et al. (2017) | Norway | Retrospective chart review | 189 patients | Hospital | Urolithiasis | CT KUB |
| Rozman et al. (2020) | USA | Retrospective chart review | 445 patients | Hospital | DVT | Lower limb US |
| Sabaté-Llobera et al. (2016) | Spain | Retrospective chart review | 28 patients | Hospital | Localized Diffuse Large B-cell lymphoma | CT |
| Salari et al. (2013) | Iran | Retrospective chart review | 300 referrals | Hospital | LBP | L-spine MRI |
| Sambandam et al. (2017) | India | Retrospective chart review | 220 patients | Hospital | Primary total knee replacement | Knee XR |
| Schlechter & Dempewolf (2015) | USA | Retrospective chart review | 532 children | Children’s hospital | Supracondylar humerus fracture | XR humerus |
| Schlemmer et al. (2015) | USA | Retrospective chart review | 14,838 cases | Emergency department | LBP | L-spine XR, CT, MRI |
| Schumacher et al. (2018) | USA | Retrospective chart review | 10,838 patients | Hospital | Breast cancer | Asymptomatic systemic imaging during follow-up |
| Scott et al. (2014) | USA | Retrospective chart review | 313 patients | Hospital | Syncope | Carotid US |
| Sener et al. (2014) | Turkey | Cross-sectional multicentric study | 535 cases | Hospital | Nasal fractures | Facial CT, XR |
| Shahi et al. (2015) | USA | Retrospective chart review | 32,432,686 children | Emergency department | Falls | CT |
| Sharma et al. (2019) | UK | Retrospective chart review | 237 patients | Hospital | Distal radius fractures | Wrist XR |
| Shau et al. (2012) | USA | Retrospective chart review | 301 patients | Hospital | C- spine fusion | C-spine XR |
| Sheehan et al. (2016) | USA | Retrospective chart review | 237 patients | Tertiary care | Shoulder pain | Shoulder MRI |
| Sheridan et al. (2020) | Ireland | Retrospective cohort study | 109 patients | Hospital | Knee pain | Knee MRI |
| Shimoni et al. (2020) | Israel | Prospective cohort study | 273 patients | Hospital | Patients without respiratory tract symptoms | Chest XR |
| Shinagare et al. (2015) | USA | Retrospective chart review | 101 patients | Emergency department | Acute pancreatitis | Abdominal CT and MRI |
| Shobeirian et al. (2020) | Iran | Descriptive prospective study | 170 patients | Emergency department | Minor head injury | Head CT |
| Shuaib et al. (2014) | USA | Retrospective chart review | 239 patients | Hospital | Shunt malfunction | Head XR head |
| Sifri et al. (2011) | USA | Retrospective chart review | 107 patients | Level 1 trauma center | Minor head injury | Consecutive head CT |
| Simos et al. (2015) | Canada | Retrospective chart review | 26,547 patients | Hospital | Breast cancer | Asymptomatic systemic imaging during follow-up |
| Simpson et al. (2013) | USA | Retrospective chart review | 146 patients | Hospital | Lumbar interbody fusion | L-spine XR |
| Sincavage et al. (2019) | USA | Retrospective chart review | 463 children | Tertiary care children’s hospital | Appendicitis | abdominal MRI |
| Singh & Jayachandran (2014) | India | Comparative study | 40 patients | Hospital | Zygomatic arch and mandibular fractures | Facial CT, XR |
| Smith et al. (2020) | Australia | Retrospective chart review | Knee MRI patients | National | Knee pain | Knee MRI |
| Sobiecka et al. (2016) | Poland | Retrospective chart review | 1,068 referrals | Hospital | Several | CT and MRI in general |
| Sola et al. (2019) | USA | Retrospective chart review | 360 patients | Hospital | Pectus excavatum | Consecutive Chest XR |
| Solivetti et al. (2013) | Italy | Retrospective chart review | 546 patients | Hospital | Cutaneous melanoma | US |
| Solivetti et al. (2016) | Italy | Retrospective chart review | 400 patients | Hospital | Knee pain | Knee MRI |
| Soydal et al. (2019) | Turkey | Retrospective chart review | 46 patients | Hospital | Prostate carcinoma | Bone scintigraphy and Ga-68 prostate-specific membrane antigen PET/CT |
| Spanier et al. (2010) | The Netherlands | Multicenter observational study | 166 patients | Hospital | Acute Pancreatitis | Abdominal CT |
| Sreedharan et al. (2014) | Tasmania | Retrospective chart review | 108 patients | Hospital | Abdominal pain | Abdominal XR |
| Stenroos et al. (2019) | Finland | Retrospective chart review | 224 children | Hospital | Fractures | Follow-up XR |
| Stippler et al. (2012) | USA | Systematic review | 1,630 patients, 19 studies | Hospital | Minor head injury | Consecutive head CT |
| Stone et al. (2015) | USA | Retrospective chart review | 268 patients | Hospital | Distal radius fractures | Follow-up wrist XR |
| Stott & Balogh (2017) | Australia | Systematic review | 31 studies | Hospital | Peri-articular fractures | Postoperative CT |
| Strait et al. (2020) | USA | Retrospective chart review | 257,661 children | Hospital | Pediatric trauma | Head, thoracic, and abdominal CT |
| Streck et al. (2017) | USA | Prospective, observational study | 2,188 children | Level 1 pediatric trauma center | Blunt trauma | Abdominal CT |
| Subramanian et al. (2016) | USA | Retrospective chart review | 125 patients | Level 1 trauma center | Near hanging | Neck CT/MRI |
| Tafazal & Flowers et al. (2015) | UK | Retrospective chart review | 119 children | Hospital | Hip dysplasia | Hip XR |
| Taghipour et al. (2017) | USA | Retrospective chart review | 433 patients | Hospital | Various cancers | Follow-up 18F-FDG PET/CT |
| Tan et al. (2016) | USA | Retrospective cohort study | 145,320 patients | Primary care | LBP | L-spine CT, MRI or XR |
| Thanh et al. (2010) | Canada | Retrospective chart review | 74,496 patients | Hospital | Elective surgery | Chest XR |
| Theisen-Toupal et al. (2014) | USA | Retrospective chart review | 398 patients | Medical center | Delirium | Head CT |
| Thiriez et al. (2015) | France | Retrospective chart review | 516 patients | Hospital | Movement disorders | [123I]-FP-CIT, SPECT |
| Thompson et al. (2014) | USA | Retrospective chart review | 680 patients | Hospital | Diffuse large B-cell lymphoma | follow-up CT, PET |
| Thornburg et al. (2017) | USA | Retrospective chart review | 197 patients | Trauma centers/rural health centers | Trauma | Whole body CT |
| Tisch et al. (2013) | Germany | Retrospective chart review | 1,000 patients | Hospital | Chronic Eustachian tube dysfunction | Inner ear CT |
| Tournemine et al. (2019) | France | Retrospective chart review | 100 children | Hospital | Adolescent idiopathic scoliosis | Total spine XR |
| Tran et al. (2013) | USA | Retrospective chart review | 354 patients | Hospital | Neck pain | C-spine XR flexion - extension |
| Trofimova et al. (2020) | USA | Retrospective chart review | 4,257 children | Hospital | Headache | Head XR, CT and MRI |
| Truong et al. (2014) | USA/France | Retrospective chart review | 173 patients | Hospital | Non-Hodgkin lymphoma | CT or PET/CT |
| Tuomilehto et al. (2017) | Finland | Retrospective chart review | 264 children | Hospital | Displaced supracondylar humerus fracture | XR humerus |
| Turnbull et al. (2010) | UK | Open, parallel group trial | 1,623 patients | Medical center | Breast cancer | MRI |
| Uccella et al. (2016) | Italy | Retrospective chart review | 3,021 patients | Emergency department | Minor head injury | Consecutive head CT |
| Uriel et al. (2017) | USA | Retrospective, cross-sectional study | 460 patients | Level 1 trauma center | Trauma | C-spine CT |
| Valusek et al. (2010) | USA | Retrospective chart review | 843 patients | Hospital | Gastroesophageal reflux disease | UGI contrast examination |
| Van den Bergh et al. (2011) | The Netherlands | Retrospective chart review | 579 patients | Hospital | Maxillofacial trauma | Facial XR |
| Van Gerven et al. (2019) | The Netherlands | Multicenter, prospective, RCT | 326 patients | Hospital | Distal radial fractures | Follow-up wrist XR |
| Van Randen et al. (2011) | The Netherlands | Prospective trail | 1,021 patients | Hospital | Abdominal pain | Abdominal XR |
| Van Trigt et al. (2018) | International | Meta-analysis | 11,423 patients | Multicenter | Asymptomatic hemodynamically stable blunt trauma | Pelvic XR |
| Vanderby et al. (2015) | Canada | Systematic review | 14 studies | Hospital | Different | MRI |
| Vejdani et al. (2015) | Iran | Descriptive study | 150 cases | Hospital | Knee pain | Knee MRI |
| Velickovic et al. (2013) | Serbia | Prospective observational study | 97 patients | Intensive care unit | Digestive Surgery | Chest XR |
| Verma et al. (2011) | USA | Retrospective chart review | 229 patients | Hospital | Admission | Chest XR |
| Vernon et al. (2015) | USA | Retrospective chart review | 76 children | Hospital | Myelomeningocele | TTE |
| Viau et al. (2019) | Canada | Systematic review | 3,361 patients, 17 studies, | Emergency department | Syncope | Head CT |
| Vijayakrishnan et al. (2015) | USA | Retrospective chart review | 400 patients | Hospital | Delirium | Head CT |
| Vilar-Palop et al. (2018) | Spain | Cross-sectional retrospective study | 2,022 examinations | Tertiary care | Several | CT, MRI, and XR |
| Voss et al. (2012) | USA | Retrospective chart review | 216 children | Pediatric Oncology Group | Hodgkin’s lymphoma | Surveillance CT |
| Wallace et al. (2016) | USA | Retrospective chart review | 169 patients | Medical center | Breast cancer | Follow-up <12 months after treatment |
| Wang et al. (2013) | China | Prospective cohort study | 183 patients | Hospital | Ankle fracture | Ankle XR |
| Wang et al. (2016) | USA | Retrospective chart review | 38,971 patients | Emergency department | Breast cancer | Breasts MRI |
| Wang et al. (2018) | China | Randomized controlled trial | 2,140 patients | Hospital | Headache | Head CT |
| Wang, Long et al. (2016) | USA | Retrospective cohort study | 9,166 patients | Hospital | Breast cancer | Breasts MRI |
| Washington et al. (2012) | USA | Retrospective chart review | 321 patients | Hospital | Minor head injury | Consecutive head CT |
| Weiss et al. (2013) | USA | Retrospective chart review | 1,687 children | Hospital | Embryonal rhabdomyosarcoma | Bone scan |
| Werner et al. (2016) | USA | Retrospective chart review | 599 patients | Hospital | Anterior cruciate ligament reconstruction | Knee XR |
| Westerterp et al. (2013) | The Netherlands | Retrospective chart review | 254 patients | Hospital | Hip fracture | Hip XR |
| Westphalen et al. (2011) | USA | Retrospective cross-sectional analysis | 3,818 patients | Emergency department | Urolithiasis | Abdominal CT/US |
| Whittam et al. (2014) | USA | Retrospective chart review | 91 children | Children’s hospital | Multicystic dysplastic kidney | Tc-99m MAG3 or DMSA |
| Wilhelm et al. (2019) | Germany | Prospective cohort study. | 38 patients | Hospital | Urolithiasis | Consecutive abdominal CT |
| Wilson et al. (2015) | USA | Retrospective chart review | 121 patients | Hospital | Long bone cartilaginous lesions | MRI, CT, bone scan, or skeletal survey |
| Witteles et al. (2012) | USA | Retrospective chart review | 78,705 patients | Hospital | Coronary disease | Ventriculography |
| Wnuk et al. (2018) | USA | Retrospective cohort study. | 5,365 patients | Hospital | LBP | L-spine MRI |
| Wong et al. (2011) | USA | Retrospective chart review | 579 children | Hospital | Blunt trauma | Pelvic XR |
| Wong et al. (2015) | Canada | Prospective observational cohort study | 169 children | Children’s hospital | Cryptorchidism | Scrotal US |
| Woodland et al. (2018) | USA | Retrospective chart review | 200 patients | Hospital | Pneumothorax | Chest XR |
| Woodward et al. (2013) | USA | Prospective cohort study | 53 children | Hospital | Pneumothorax | Chest XR |
| Wright et al. (2015) | USA | Systematic review | 21 studies | Hospital | Stress fractures | Scintigraphy, XR, US and CT |
| Wrotek et al. (2019) | Poland | Retrospective chart review | 581 children | Hospital | Bronchiolitis | Chest XR |
| Wu et al. (2013) | Taiwan | Retrospective chart review | 103 patients | Hospital | Esophageal perforation | fluoroscopic esophagography |
| Wu et al. (2017) | China | Retrospective chart review | 108 patients | Hospital | Acute appendicitis | Abdominal CT |
| Wymer et al. (2017) | USA | Retrospective chart review | 593 patients | Medical center | Testicular Cancer | PET, Brain imaging, Bone scan |
| Yamashita et al. (2011) | USA | Retrospective chart review | 63 patients | Hospital | Lumbar fusion | L-spine XR |
| Yıldızhan et al. (2019) | Turkey | Retrospective chart review | 43,389 patients | Emergency department | Head injuries | Head CT |
| Yonis et al. (2019) | Several | Systematic review | 16 studies | Hospital | Anastomotic leak post esophagectomy | Esophagus XR |
| Yoo et al. (2019) | USA | Retrospective chart review | 188 patients | Hospital | DVT | Venous duplex ultrasound |
| You et al. (2011) | Canada | Retrospective chart review | 623 patients | Ambulatory care | Headache | Head CT |
| Yu et al. (2016) | China | Retrospective chart review | 3,107 examinations | Hospital | LBP | L-spine MRI |
| Yun et al. (2018) | USA | Survey | 99,135 ED visits | Emergency department | Head injury | Head CT |
| Zagory et al. (2017) | USA | Retrospective chart review | 247 children | Level 1 pediatric trauma center | Blunt trauma | Abdominal CT |
| Zargar Balaye Jame et al. (2014) | Iran | Cross-sectional study | 400 patients | Imaging centers | Minor Head Injury | Head CT |
| Zargar et al. (2014) | Iran | Cross‑sectional study | 400 patients | Imaging centers | LBP | L-spine MRI |
| Zeechan et al. (2019) | USA | Retrospective chart review | 423 children | Hospital | Liver injury | Abdominal CT |
| Zhou et al. (2020) | China | Retrospective cohort study | 29 patients | Hospital | Covid-19 | Consecutive chest CT |
| Zhu et al. (2014) | China | Prospective cohort study | 455 children | Hospital | Head trauma | Head CT |
| Zieleskiewicz et al. (2018) | France | Retrospective single center study. | 756 patients | Level 1 trauma center | Sever trauma | Chest and pelvic XR |
| Zulfiquar et al. (2017) | USA | Retrospective chart review | 316 children | Hospital | Skull fracture | Consecutive head CT |
| Zuzek et al. (2019) | Slovenia | Retrospective chart review | 500 referrals | Emergency department | Head injury | Head CT |
